# Low-Cost, Large-Scale Production of the Anti-viral Lectin Griffithsin

**DOI:** 10.3389/fbioe.2020.01020

**Published:** 2020-08-21

**Authors:** John S. Decker, Romel Menacho-Melgar, Michael D. Lynch

**Affiliations:** Department of Biomedical Engineering, Duke University, Durham, NC, United States

**Keywords:** griffithsin, biologics manufacturing, downstream recovery, antiviral, SARS-CoV-2

## Abstract

Griffithsin, a broad-spectrum antiviral lectin, has potential to prevent and treat numerous viruses including HIV, HCV, HSV, SARS-CoV, and SARS-CoV-2. For these indications, the annual demand for Griffithsin could reach billions of doses and affordability is paramount. We report the lab-scale validation of a bioprocess that supports production volumes of >20 tons per year at a cost of goods sold below $3,500/kg. Recombinant expression in engineered *E. coli* enables Griffithsin titers ∼2.5 g/L. A single rapid precipitation step provides > 90% yield with 2-, 3-, and 4-log reductions in host cell proteins, endotoxin, and nucleic acids, respectively. Two polishing chromatography steps remove residual contaminants leading to pure, active Griffithsin. Compared to a conventional one this process shows lower costs and improved economies of scale. These results support the potential of biologics in very large-scale, cost-sensitive applications such as antivirals, and highlight the importance of bioprocess innovations in enabling these applications.

## Highlights

1. High titer production of Griffithsin in *E. coli.*
2. Low cost purification of Griffithsin enabled by precipitation.
3. Scalable, cost effective manufacturing process for a potential broad spectrum antiviral.

## Introduction

**FIGURE 1:**
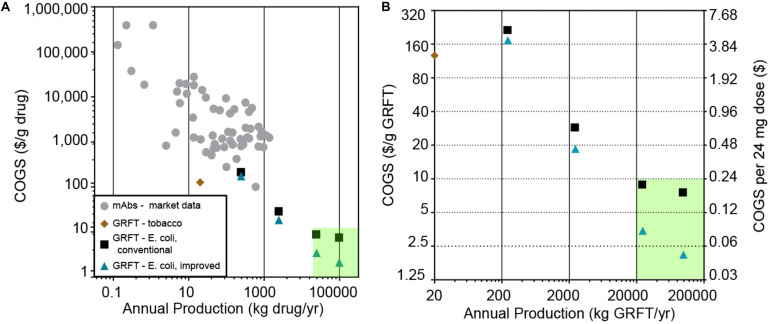
**(A)** Estimated cost of goods sold and scale of production for various protein drug products. Gray circles: monoclonal antibodies (mAbs), based on an analysis of publicly available financial data summarized in Supplementary Table S2. Brown diamond: results of an *in silico*-modeled tobacco-based process for Griffithsin (GRFT) by Alam et al. (2018), which does not include formulation or packaging costs. Black squares: *in silico*-models of a GRFT process based on *E. coli* fermentation and a conventional, chromatography-based downstream purification, modeled at various scales. Blue triangles: *in silico* models of GRFT processes based on *E. coli* fermentation and a downstream purification based on the precipitation step reported here, modeled at various scales. The green shaded area indicates an estimated target range for large-scale, low-cost deployment of an anti-SARS-CoV-2 biologic (>20,000 kg/yr at <$10.00/g). **(B)** The GRFT processes and target region from **(A)**, replotted on zoomed-in axes for clarity. Cost per 24 mg dose is also shown, assuming packaging in 200 mg multi-dose vials.

There is a significant need for broad-spectrum antiviral drugs that are effective, safe, widely available and affordable, as has been highlighted by the current COVID-19 pandemic. Among the drugs currently being developed against SARS-CoV-2 are many proteins (biologics), especially monoclonal antibodies (mAbs) (Li and De Clercq, 2020). While these drugs hold great promise for efficacy and safety, as a class biologics are costly to manufacture (Farid, 2017; Figure 1A, and Supplementary Table S2), costly for patients and insurers (Blackstone and Fuhr, 2007), and usually produced on relatively small scales compared to traditional drugs. Therapeutic mAbs are typically produced on a scale of only hundreds of kg/yr or less, with manufacturing costs (costs of goods sold, COGS) of >$100/g (Figure 1A, Supplementary Table S2) and sales prices of >$1000/g (Supplementary Table S2). In contrast, assuming a typical biologic drug dose of tens to hundreds of milligrams and a global demand for hundreds of millions of doses for a biologic antiviral against a pandemic virus, we estimate a need for production volumes of tens of thousands of kg/yr and COGS of less than $10/g.

One factor that tends to sharply lower COGS in manufacturing is increasing production scale (Figure 1), thanks to economies of scale. However, in the production of biologics there are meaningful limitations to current economies of scale. According to one estimate, mAb production costs have the potential to be as low as $20/g if scaled up to production levels of 10,000 kg/yr, which is approximately 10 times larger than the largest-scale mAb process commercialized to date (Kelley, 2009). Insulin, likely the least expensive and largest-scale biologic, has a global patient population of approximately 100 million and a production volume on the order of 50,000 kg per year, with an estimated COGS still above $25/g before formulation (Gotham et al., 2018). Therefore it is unclear whether economies of scale in current biologics manufacturing processes are sufficient to allow COGS significantly below $20–25/g at any scale.

These limitations call into question whether widespread use of biologics against SARS-CoV-2 or other pandemic viruses will be commercially feasible, especially in more resource-limited settings (Saqib et al., 2018; Ewen et al., 2019). Thus, even while safety and efficacy remain to be established for anti-SARS-CoV-2 biologic candidates, finding ways to increase accessibility and reduce costs for these drugs is also paramount and should proceed in parallel with drug validation. Toward this end, we report improved manufacturing techniques for the promising broad-spectrum antiviral protein Griffithsin (GRFT).

GRFT and its derivatives (e.g., the oxidation-stable variant Q-GRFT) (Günaydın et al., 2019) are small lectins originally isolated from the red algae *Griffithsia* (Mori et al., 2005). GRFT binds and neutralizes many enveloped viruses including HIV (O’Keefe et al., 2009), HSV and HPV (Derby et al., 2018), HCV (Meuleman et al., 2011), and SARS-CoV (O’Keefe et al., 2010). GRFT’s selectivity for high-mannose glycans (Moulaei et al., 2010; Xue et al., 2013), which are host-derived and widely conserved on the surface proteins of enveloped viruses (Vigerust and Shepherd, 2007; Bagdonaite and Wandall, 2018), make it a broad-spectrum entry inhibitor and potentially a neutralizer of many emerging viruses (van der Meer et al., 2007; O’Keefe et al., 2010; Sato et al., 2011). This protein has undergone clinical evaluation (including Phase 1 clinical trials) as a microbicide to prevent HIV transmission during sexual intercourse (US National Library of Medicine, 2020, Study to Evaluate the Safety of Griffithsin in a Carrageenan Gel in Healthy Women) and has shown an excellent safety profile in animals including non-human primates when applied topically, intravenously or subcutaneously (O’Keefe et al., 2009; Ishag et al., 2013; Nixon et al., 2013; Barton et al., 2014; Kouokam et al., 2016; Derby et al., 2018).

Although GRFT has not previously been shown to neutralize SARS-CoV-2, it is a promising candidate: the SARS-CoV-2 spike protein is known to have a high degree of glycan conservation with the SARS-CoV spike protein, (Walls et al., 2020) and GRFT has been shown to be safe and effective in preventing SARS-CoV-related mortality and morbidity when administered intranasally to mice (O’Keefe et al., 2010). While additional studies are needed to determine the efficacy and safety of GRFT for prevention or treatment of SARS-CoV-2, significant manufacturing challenges must also be solved in parallel. Based on the previous studies in mice for SARS-CoV, where doses of 2.5–5 mg/kg-day were evaluated, (O’Keefe et al., 2010) allometric scaling would suggest an effective human dose in the range of 0.2–0.4 mg/kg-day or ∼12–24 mg/day or more (Nair and Jacob, 2016). As at least an approximate starting point for a human dose, this suggests that meeting the potential global demand for an effective antiviral against SARS-CoV-2 would require at least thousands of kg of GRFT and would cost hundreds of millions or billions of dollars even if priced at the extreme low end of the typical range for biologics. Even broader deployment, such as for a prophylactic, would require tens of thousands of kg of GRFT.

In this work, we develop a proof of principle bioprocess that supports GRFT production at dramatically larger scales and lower COGS than previously demonstrated. Importantly, this process does not just reduce COGS by some fixed proportion, but shows improved economies of scale so that its advantage continues to increase as production scale rises. This is achieved by replacing a costly and poorly scaling chromatography operation in the downstream purification (DSP) with a precipitation step. We estimate that this process would enable a single plant, with 60,000 L of fermentation capacity and approximately $130 million in fixed capital costs, to produce >24,000 kg GRFT/yr at a COGS as low as $3.43/g.

## Materials and Methods

### Reagents and Media

SM10, AB, and AB-C7 media were prepared as previously reported (Menacho-Melgar et al., 2020b). Kanamycin sulfate was used at a working concentration of 35 μg/mL. Unless otherwise stated, all materials and reagents were of the highest grade possible and purchased from Sigma (St. Louis, MO).

### Strains and Plasmids

*Escherichia coli* strain DLF_Z0025 was constructed as previously reported (Li et al., 2020; Menacho-Melgar et al., 2020b). The GRFT sequence as reported by Mori et al. (Mori et al., 2005) was codon-optimized and incorporated in a synthetic DNA construct (IDT, Coralville, IA) under the control of the low phosphate inducible *phoA, phoB*, and *yibD* gene promoters (Moreb et al., 2020). These genes were inserted into the pSMART-HC-Kan vector (Lucigen, Middleton, WI) using 2X HiFi DNA Assembly Master Mix from New England Biolabs (Ipswich, MA) according to manufacturer instructions. The resulting plasmids were pHCKan-phoAp-GRFT (Addgene #158747), pHCKan-yibDp-GRFT (Addgene # 158745), pHCKan-phoBp-GRFT (Addgene # 158746). Additionally, a plasmid to express Q-GRFT [an enhanced variant of GRFT (Günaydın et al., 2019)], was cloned using around the world Q5 mutagenesis (New England Biolabs, Ipswich, MA) following manufacturers instruction, pHCKan-yibDp-GRFT as a template and primers Q-GRFT_F and Q-GRFT_R (GGCCCATACGGAGGGTCG and GAAACGACGCCCCTGATTCGTCTC, respectively). The resulting plasmid was pHCKan-yibDp-Q-GRFT (Addgene # 158748). All plasmids were confirmed via Sanger sequencing at Genewiz, Research Triangle Park, NC.

### Fermentations

Microfermentations (using AB media), shake flask expression (using SM10 or AB-C7 media) and instrumented fermentations were performed as previously described (Menacho-Melgar et al., 2020b).

### Technoeconomic Analysis and *in silico* Bioprocess Modeling

Models of GRFT production bioprocesses were created in SuperPro Designer (Intelligen, Inc., Scotch Plains, NJ). Each process was designed to produce GRFT meeting typical biologics standards: virus-free, <10 ng residual cellular DNA (rcDNA)/dose (World Health Organization, 1998), (<100 pg/mg GRFT assuming a maximum dose of 100 mg), <100 ppm host cell proteins,(Wang et al., 2009) and endotoxin levels less than 12.5 endotoxin units (EU) per mg GRFT (assuming a limit of 5 EU per kg patient bodyweight (The United States Pharmacopeial Convention, 2011) and a dose of 0.4 mg/kg). Default options for SuperPro’s built-in cost models were used to estimate expenses including equipment, consumables, other capital investment, insurance and taxes, maintenance, labor and utilities. A 10 year straight line depreciation model was used for all equipment. The plant was assumed to be operational for 85% of each year. Sensitivity analyses (Figure 3) were conducted by changing relevant process variables one at a time, and scaling analyses (Figure 1) were conducted by linearly scaling equipment capacities and adjusting materials costs according to an exponential economies of scale model. For further details on modeling, refer to Supplementary Material.

### Design of Experiments and Precipitation Optimization

Design of experiments (DoE) was used to facilitate the optimization of precipitation conditions for GRFT purification through two rounds of experiments. In the first screening round, a Definitive Screening Design (JMP^§^, Version 14. SAS Institute, Cary, NC) was used with two outcomes, GRFT separation factor and yield, and four factors: incubation temperature and time, pH, and (NH_4_)_2_SO_4_ concentration. In a second round guided by the results of the first, a central composite design was used with the addition of protein concentration as a factor and the removal of time and (NH_4_)_2_SO_4_ concentration as factors. Predictive models were constructed using standard least-squares linear regression.

DLF_Z0025 containing pSMART-phoAp-GRFT was cultured in shake flasks as previously described (Menacho-Melgar et al., 2020b). Cells were harvested by centrifugation and pellets were stored at −60°C until lysis. Cell pellets were resuspended in 50 mM phosphate buffer, pH 7.2, to a density of approximately 300–400 OD_600_ and supplemented with Halt protease inhibitor (Thermo Fisher Scientific, Waltham, MA). Cells were lysed at 4°C using a sonicator with 2 mm probe operated at 45% power for 48 cycles of 15 s on, 45 s off. Lysate was cleared by centrifugation at 4°C and total protein concentration was measured using the Pierce Coomassie Plus Bradford Assay Kit (Thermo Fisher Scientific, Waltham, MA) and normalized to twice the desired protein concentration for each precipitation condition. A lysate standard for yield quantification was prepared by diluting to 0.25 g/L in lysis buffer, adding Laemmli sample buffer (Bio-Rad Laboratories, Hercules, CA), heating at 95°C for 5 min, and storing at −20°C.

Precipitation buffers were prepared in 200 μL PCR tubes by combining ultrapure water, saturated (NH_4_)_2_SO_4_ solution, and concentrated HCl or NaOH to achieve the desired pH and degree of (NH_4_)_2_SO_4_ saturation in 100 μL. Clarified lysate was added to each buffer at a 1:1 volume ratio to achieve the desired protein concentration in 200 μL, and samples were incubated in thermocyclers or dry heat blocks for the times and temperatures prescribed by each DoE condition. Immediately after each incubation, samples were harvested and clarified by centrifugation. Because the yield of GRFT in each supernatant was unknown until SDS-PAGE analysis, supernatants were diluted in lysis buffer such that a 50% yield of GRFT would result in equal GRFT concentrations between a given sample and the lysate standard (e.g., samples with initial concentrations of 5 g/L total protein were diluted 10x; 2.5 g/L by 5x; etc.). Diluted supernatants were denatured in Laemmli buffer and stored at −20°C.

Separation factors and yields for GRFT from each precipitation condition were analyzed by densitometry of SDS-PAGE gels. 15-well NuPAGE Bis-Tris gels (Thermo Fisher Scientific, Waltham, MA) were loaded with 10 μL per well of each sample (including 5 μL Laemmli buffer) and run in MOPS buffer at 200 V. Each gel included a lysate standard from the same batch of lysate as the purified samples on that gel, to allow yield calculations. Gels were stained with SYPRO Ruby Protein Gel Stain (Thermo Fisher Scientific, Waltham, MA) according to manufacturer instructions and imaged through a 635 ± 35 nm bandpass filter under UV illumination. Densitometry was conducted using FIJI (Schindelin et al., 2012; Rueden et al., 2017). Rolling ball background subtraction was applied with a ball radius of 400 pixels (>2x the largest band dimension), and bands were automatically identified and integrated using FIJI’s built-in thresholding tool based on the method of Huang and Wang (1995) Separation factors were calculated as the ratio of GRFT to contaminants in a sample divided by the corresponding ratio in the lysate standard. Yield was calculated as the dilution-corrected ratio between the GRFT signal in a purified sample and in the lysate standard. The amount of GRFT in each sample and standard was within the linear range of the stain.

### Anion Exchange FPLC for Endotoxin Removal

Supernatants from the optimal precipitation step were pooled, exchanged by diafiltration into 100 mM pH 2.73 citrate buffer containing 50 mM ammonium sulfate (sample buffer) and stored at 4°C prior to chromatography. Strong anion exchange FPLC was performed in flowthrough mode using a 5 mL HiTrap^§^ Q FF column on an AKTA Pure instrument (GE Healthcare Life Sciences, Marlborough, MA). The flow path and column were equilibrated with 8 column volumes of sample buffer, followed by manual washing and equilibration of the 500 μL sample loop, injection of 1000 μL of sample at a flow rate of 10 mL/min, and a further wash with sample buffer at a flow rate of 10 mL/min. A single peak was observed to flow through immediately and was collected for analysis.

### rcDNA and Endotoxin Quantification

Endotoxin was quantified using the Pierce Chromogenic Endotoxin Quant Kit (Thermo Fisher Scientific, Waltham, MA) according to manufacturer instructions. rcDNA was assessed by agarose gel electrophoresis with ethidium bromide staining.

### SPR

The activity of precipitation-purified GRFT was assayed by measuring its binding to gp140 using a Biacore T200 surface plasmon resonance instrument with a CM5 sensor chip (GE Healthcare Life Sciences, Marlborough, MA). Recombinant gp140 from the clade C strain 92BR025 was obtained from Sino Biological, Inc., (catalog # 40251-V02H; Beijing, China) and immobilized to approximately 300 response units via NHS-EDC coupling. The immobilized surface was quenched with ethanolamine and equilibrated with PBS at 50 μL/min for several hours. GRFT was prepared in triplicate dilution series at concentrations of 400, 200, 100, 50, and 12.5 nM, and kinetic titrations were performed. As a positive control, the first replicate series of GRFT injections was interleaved with concentration-matched injections of purified 6x-histidine-tagged GRFT obtained from Barry O’Keefe at the National Cancer Institute. Between each sample injection, the surface was regenerated with 10 mM glycine, pH 1.5, at 50 μL/min for 15 s and then re-equilibrated with PBS at 50 μL/min for 2 min. Binding responses were adjusted by subtracting both the response observed on a blank (activated and quenched) sensor surface and the response to zero-concentration samples. For His-GRFT, *n* = 1 for all concentrations; for precipitation-purified GRFT, *n* = 3 except for 12.5 nM, for which the second replicate showed no binding and was discarded. Kinetic constants for both precipitation-purified GRFT and His-GRFT were determined using BIAevaluation software (GE Healthcare Life Sciences, Marlborough, MA) by fitting a heterogeneous ligand model to each replicate dataset independently, then calculating means and standard deviations in the case of precipitation-purified GRFT.

## Results

### A Critical Evaluation of GRFT Bioprocess Alternatives

We first sought to critically evaluate the capabilities of various potential GRFT bioprocesses, to achieve volume and cost targets as mentioned above (a COGS < $10/g and production of >5000 kg per year). These included previously demonstrated processes using engineered plants and more traditional fermentation based processes (Figure 1; O’Keefe et al., 2009; Fuqua et al., 2015a, b; Alam et al., 2018). For plant-based bioprocesses, we analyzed the impact of the specific production of GRFT (g GRFT/g plant biomass), plant biomass costs, and the scalability of plant based protein production on manufacturing volumes and costs, resulting in an estimate for the potential of plant-based processes if scaled (refer to Figure 1 and Supplementary Materials). As can be seen from this analysis, plant-based manufacturing is highly unlikely to reach target production volumes or costs for a large-scale GRFT antiviral. We then assessed the potential of more traditional fermentation-based processes (e.g., using engineered *E. coli*) modeled using SuperPro Designer (Toumi et al., 2010; Petrides et al., 2014). We based these processes on our own lab-scale results with GRFT expression and purification (see below), standard bioprocess operations adapted as appropriate, and previously reported formulations for nebulized proteins (Steckel et al., 2003; Albasarah et al., 2010). As seen in Figure 1, large-scale fermentation-based processes have the potential to produce the needed volumes of GRFT at costs within our target range.

### GRFT Bioprocess Cost Reductions

**FIGURE 2:**
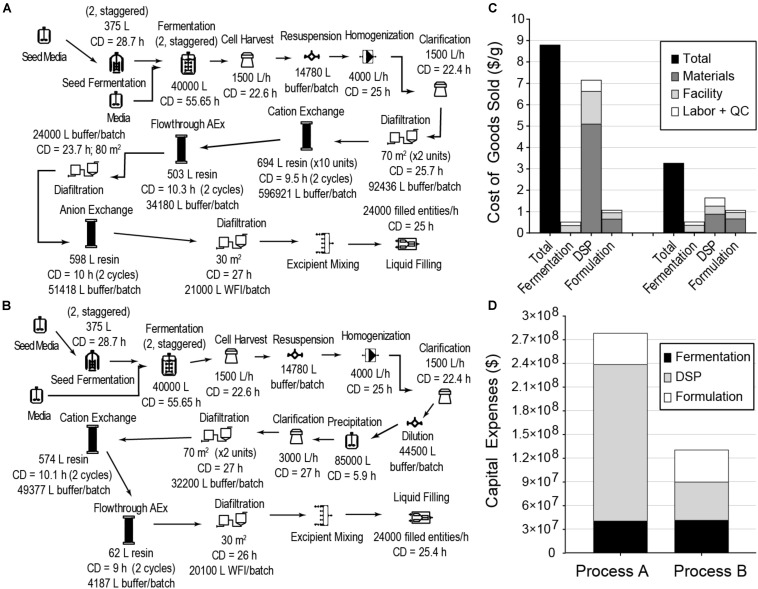
*E. coli* fermentation-based processes for GRFT antiviral manufacturing, modeled to produce approximately 24,000 kg of GRFT per year in a filled and finished antiviral (here, a multidose vial with a formulation suitable for nebulization). **(A)** Process with conventional chromatography-based purification. **(B)** Process with precipitation-based purification. **(C)** Costs of Goods Sold per gram of formulated and packaged GRFT for the processes from **(A)** (left) and **(B)** (right), shown by process section and cost category. **(D)** Estimated capital expenses for the processes from **(A,B)**, shown by process section. CD - cycle duration, AEx - Anion exchange, WFI = water for injection, DSP - downstream purification, including primary recovery, Entity - final packaged product (e.g., a filled vial). Fermentation includes seed train and primary fermentation operations. Materials include all raw materials (e.g., chemicals, water) and consumables (e.g., membranes and resins).

We next evaluated cost reduction opportunities for a fermentation-based process using a typical DSP strategy based on multiple chromatography columns and a typical formulation suitable for nebulization (Process A). This process, with 60,000 L fermentation capacity producing 24,000 kg of GRFT per year, is illustrated in Figure 2A. As can be seen in Figures 2C,D, the COGS and capital expenses (CAPEX) for this process are dominated by DSP. These results are broadly consistent with observations for most large-scale bioprocesses (Straathof, 2011). While this process using a conventional DSP can reach target GRFT production scales (>20 tons annually), the conventional DSP approach leaves significant opportunities for cost reduction.

**FIGURE 3:**
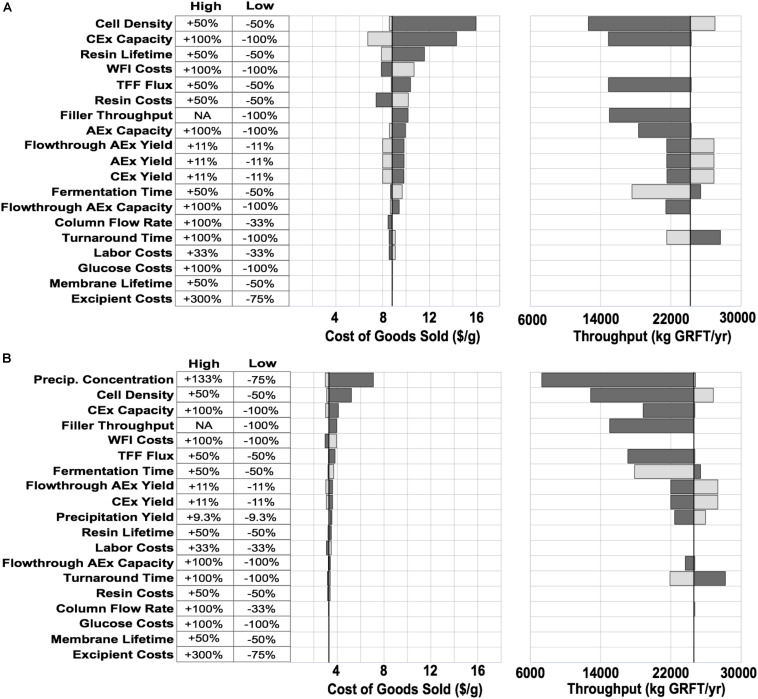
COGS and throughput sensitivities for the two processes shown in Figure 2. Variables along the Y-axis were varied one at a time and in each case the model was then adjusted to maximize throughput and minimize cost without any changes in equipment or plant layout. Light gray bars show high conditions (increasing the variable), dark gray bars show low conditions (decreasing the variable). Black line shows baseline values for each model. **(A)** Conventional purification. **(B)** Precipitation-based purification. Filler - the vial filler used in formulation. AEx - anion exchange, CEx - cation exchange, TFF - tangential flow filtration.

To reduce overall process costs, we began by targeting the key drivers of DSP costs in Process A: buffer and resin consumption (L/unit GRFT), and especially the first chromatography step. Operations related to this step account for more than 60% of total process COGS. Of this fraction, buffers and resin make up approximately 35% and 42%, respectively, while facility-related costs account for 20% and are dominated by the chromatography columns but also include a significant contribution from buffer storage tanks. In this first chromatography step the feed stream is least pure and the loading of resin and buffer is therefore least efficient. Encouraged by GRFT’s known thermostability and acid tolerance, (O’Keefe et al., 2009) we considered the potential of a precipitation step to quickly remove most contaminants with minimal materials and consumables costs. Based on pilot precipitation studies in our lab, we modeled a process (Process B) using the same upstream and formulation operations as Process A but with a DSP consisting of one precipitation step and two chromatography steps. This process is shown in Figure 2B. As can be seen in Figure 2C (Process B) and Figure 1, if the first chromatography step from Process A could be replaced with a precipitation step, COGS could be greatly reduced to below $3.50/g. This is accomplished primarily through a reduction in DSP materials costs, though they remain the largest contributor to DSP costs. A sensitivity analysis of this process (Figure 3B) revealed that reducing the total protein concentration during the precipitation increases costs as buffer consumption increases, while further increases (from our baseline concentration of 20 g/L) lead to only modest cost reductions as material costs become a smaller fraction of total process costs. Interestingly, when holding equipment constant, this process is less sensitive than Process A to decreases in the fermentation cell density (or other decreases in GRFT titer), because decreases in titer leave Process A with oversized columns that still consume as much buffer and resin as before while processing less GRFT. In contrast, in Process B, the feed stream entering the precipitation step can easily be concentrated so that the material consumption per unit GRFT in precipitation remains unchanged.

Replacing a single chromatography step with a precipitation step significantly reduces COGS for cGMP GRFT production, especially at large scales. This process could enable a single plant with 60,000 L of fermentation capacity to support the manufacture of > 24,000 kg of GRFT antiviral annually, with fixed capital costs of $130 million and COGS < $3.50/g.

### Production of GRFT in Engineered *E. coli*

**FIGURE 4:**
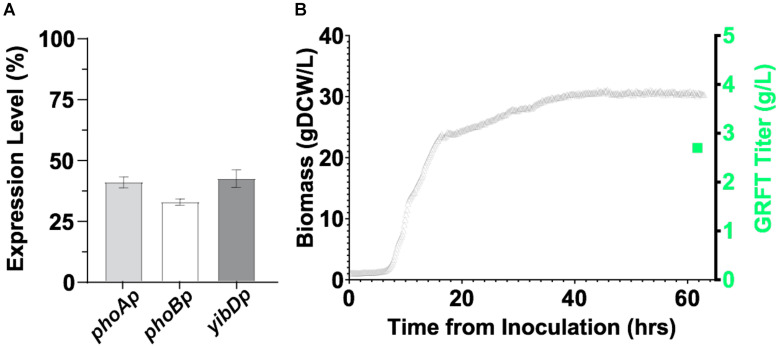
**(A)** Expression of GRFT in 96-well plate microfermentations using *E. coli* strain DLF_Z0025 containing GRFT under the control of the low-phosphate inducible promoters *phoA*, *phoB*, and *yibD*. **(B)** Expression of GRFT in a two-stage fermentation. *E. coli* strain DLF_Z0025 containing GRFT under the control of the low-phosphate inducible *phoA* promoter was cultured in a 1 L bioreactor. Biomass levels are shown by gray triangles and the final GRFT titer (∼2.7 g/L) is shown by a green square, corresponding to a GRFT expression level of ∼20% of total cell protein.

While GRFT has been previously expressed in *E. coli* in fermentations, only modest titers of ∼0.5 g/L were obtained (Giomarelli et al., 2006). First, we expressed GRFT to relatively high titers in a minimal media fermentations. This was accomplished leveraging two-stage production processes as previously reported by Menacho-Melgar et al. (2020b) and robust low phosphate inducible promoters as reported by Moreb et al. (2020). Using this platform, GRFT is expressed upon entry into stationary phase, (Burg et al., 2016) when batch phosphate is depleted. We screened three low phosphate inducible promoters (*phoA, phoB* and *yibD*) for two-stage expression in microfermentations (Figure 4A) and then scaled the production of the *phoA* promoter construct into an instrumented bioreactor producing ∼30 gCDW/L of biomass and >2.5 g/L of GRFT in a 60 h fermentation (Figure 4B). Refer to Supplementary Table S1 for plasmids and strains used in this study. Additionally, we initially validated expression of a Q-GRFT construct in microfermentations (Supplementary Figure S4).

### Optimization of Downstream Purification

**FIGURE 5:**
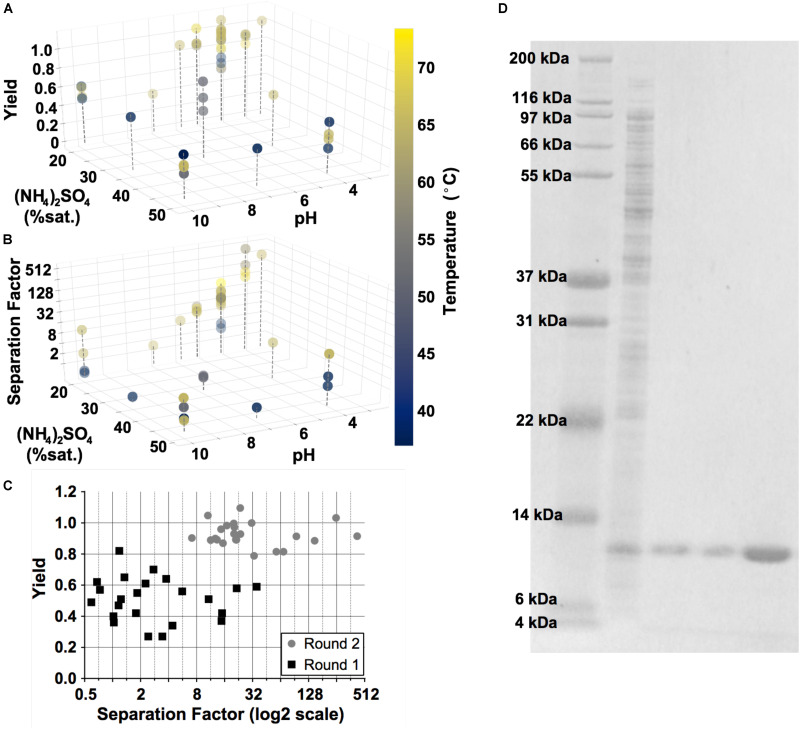
Results of Design of Experiment (DoE) studies to optimize the precipitation step. Three key variables are shown: temperature, ammonium sulfate concentration (% saturation) and pH. Two outputs were evaluated: yield **(A)** and separation factor **(B)**. Gray dashed lines are included for perspective. **(C)** A summary of these outputs over each experiment in the two rounds of DoE. **(D)** Fluorescently stained SDS-PAGE gel, converted to grayscale and with brightness values inverted for clarity. Lane 1, Ladder; Lane 2, untreated *E. coli* lysate containing GRFT (diluted 1:40); Lanes 3 and 4, supernatant following precipitation and supernatant following diafiltration into chromatography running buffer (each diluted 1:30); Lane 5, flow-through fraction from the final endotoxin removal chromatography step (diluted 5:8).

To optimize the DSP for GRFT, we leveraged standard Design of Experiments (DoE) methodology with a focus on three key variables: pH, salt concentration (NH_4_)_2_SO_4_ and temperature (Lorenzen and Anderson, 1993; Jones and Nachtsheim, 2013). GRFT was produced in shake flask cultures according to Menacho-Melgar et al. (2020b). Yield and purity were analyzed by SDS-PAGE and densitometry (refer to Materials and Methods), as shown in Figure 5. Elevated temperature, low pH and relatively low (NH_4_)_2_SO_4_ concentrations were found to be optimal. In the best conditions, we achieved a greater than 99% purity by fluorescently stained SDS-PAGE and a yield of 91.5%. In addition, precipitation reduced rcDNA to below detectable levels (Supplementary Figure S2) and endotoxin levels by approximately 1000-fold compared to untreated lysate (not shown). Residual endotoxin was removed by a single flow through anion exchange chromatography step, as described in Materials and Methods, leading to endotoxin levels less than 1.0 endotoxin units (EU) per mg GRFT, below anticipated FDA limits (Center for Drug Evaluation and Research, 2019). This process was subsequently validated with GRFT produced from 1-L fermentations.

### Precipitation-Purified GRFT Retains Anti-gp140 Activity

**FIGURE 6:**
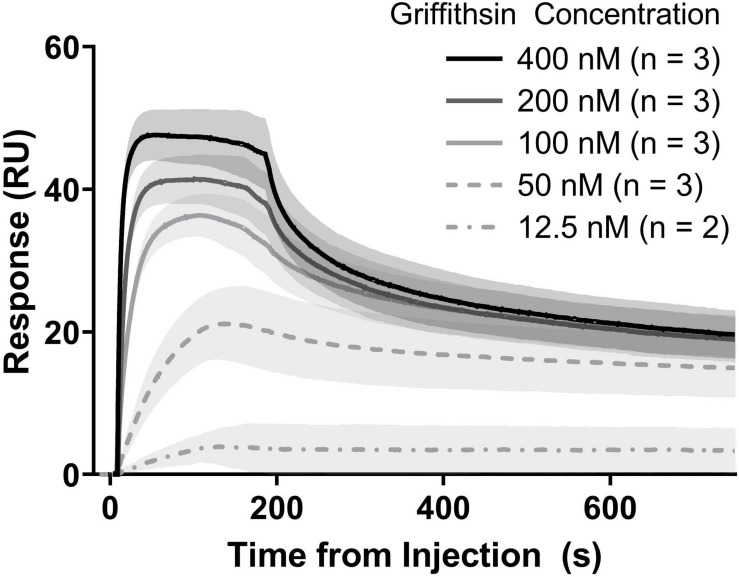
Binding kinetics of purified GRFT vs. purified HIV gp140. Measurements were accomplished with SPR. Lines show the mean response at each concentration and shaded areas show standard deviations. Each replicate set was analyzed independently by fitting with a heterogeneous ligand model, followed by calculation of summary statistics.

We next turned to evaluating our precipitation-purified GRFT’s performance in a test of *in vitro* binding to recombinant glycosylated HIV gp140 (subtype C, strain 92BR025). Binding kinetics were measured using surface plasmon resonance (SPR) (Figure 6). K_*on*_ (association rate) and K_*off*_ (dissociation rate) were measured to be 1.3 ± 0.6 × 10^7^ and 6.4 ± 5.2 × 10^–2^ respectively, resulting in an affinity of 4.4 ± 1.6 × 10^–9^ M. These measurements agreed well with previously reported measurements using GRFT and the closely related gp140 of strain Du151, (Fuqua et al., 2015a) as well as with our own results using poly histidine tagged GRFT purified via immobilized metal affinity chromatography (Supplementary Figure S3).

## Discussion

Producing a biologic at the costs and scales relevant to a pandemic response presents a novel and significant challenge, in addition to establishing a drug candidate’s safety and efficacy. To address this challenge, we have developed a process for production of the broad-spectrum antiviral GRFT (Figure 2B), which we estimate would allow a $130 million plant to produce GRFT at scales of > 24,000 kg/yr and COGS as low as $3.43/g. Based on an estimated dose of 12–24 mg, this would be sufficient to provide on the order of 1 billion doses/yr at a COGS of about $0.08/dose. Though work remains to further validate both the production process and importantly GRFT as a drug candidate, these results support the feasibility of developing biologics for large-scale, cost-sensitive applications such as antivirals.

These results also suggest a general strategy for other large-scale biologics production processes. Conventional processes have difficulty reaching extremely low COGS in part because their costs at large scale become dominated by downstream operations that have inherently poor economies of scale, especially chromatography (Figure 2C, Process A). This results in COGS reaching a plateau at sufficiently large scales (Figure 1B, Process A). The process reported here highlights one solution to that problem. Replacing only the first of three chromatography operations with a precipitation step not only reduces material and facility costs per kg of GRFT purified, but also shifts costs away from items that tend to scale poorly and toward those that tend to scale well: e.g., from columns to stirred tanks and from resins to bulk chemicals and buffers. This allows COGS for the precipitation-based process to continue declining sharply even up to scales approaching 100,000 kg/yr (Figure 1B, Process B).

### Future Work in the Development of GRFT as a Large-Scale Antiviral

Though large-scale manufacturing of an affordable biologic antiviral presents its own challenge, there is of course also an urgent need to validate GRFT’s safety and efficacy against SARS-CoV-2 and other viruses. This will require defining formulations and dosages as well as preparing sufficient material for preclinical and clinical trials. A final GRFT dosage and formulation are significant unknowns that could affect the process economics reported here. Our model assumes a typical nebulized protein formulation and packaging in 200 mg multi-dose vials in order to capture some approximate formulation costs, though the final formulation and packaging will likely be different. Drug masses per vial much larger or smaller than anticipated may affect the throughput of fill-finish operations as well as the costs of packaging, both of which are significant cost drivers in the optimized process (Figure 2C). Most importantly, any need to produce a solid form such as a lyophilized powder for reconstitution would have a substantial impact on costs for a large-scale process because of the low throughput and poor scalability of lyophilization. Thus, GRFT variants such as Q-GRFT that have enhanced stability in solution may be particularly important for further study.

To prepare sufficient GRFT for any clinical trials, our process would have to be transferred from the lab scale to facilities with hundreds of liters of fermentation capacity. The most significant unknowns in this regard are the scale-up of the precipitation step and the subsequent clarification steps. While principles for scale-up of precipitation are well known (Martinez et al., 2019), the process is not necessarily trivial. Though at the lab scale we observe large precipitate particles that are easily cleared, the sheer mass of solids to be removed in a large-scale process as well as the potential for the particle size distribution to change with scale-up may necessitate some process optimization.

If the process developed here will be implemented at large scale, it is worth considering whether other changes can bring further improvements. A sensitivity analysis (Figure 3B) did not indicate significant opportunities for COGS reductions in the large-scale model. On the other hand, this simple analysis cannot capture all the complexities and interdependencies of a pharmaceutical process. The two most promising possibilities may be increases in GRFT expression or the use of engineered host strains that reduce DNA in lysates (Menacho-Melgar et al., 2020a). Either of these changes may reduce the number of polishing operations required after precipitation. Nonetheless, the impact of these operations is much less than the cost of the first chromatography operation replaced by precipitation, and the process costs are already approaching being dominated by formulation operations (Figure 2C).

### Precipitation as a Cost-Effective Alternative to Chromatography

Beyond GRFT, this work supports the potential of precipitation to replace some chromatography operations in biologic drug manufacturing. The benefits of such a change can be very significant, and increase with increasing process scale. Especially important are operations that can replace the earliest chromatography steps in the downstream process: these steps tend to have the highest costs because they have the highest loading of contaminants and therefore the least efficient use of buffer, resin, and installed column capacity. Thus, ideal precipitations for reducing costs would be capable of delivering at least crudely pure product out of the highly impure feed streams present at the start of DSP, while conserving buffer, equipment capacity, and unit operations by working with high protein concentrations and leaving the product soluble.

The specific precipitation demonstrated here uniquely leverages the unusual thermostability and acid stability of GRFT. This helps the optimized precipitation step to achieve levels of purity and yield comparable to the most effective chromatographic operations, including Protein A chromatography for mAbs. However, even a less effective precipitation step may still provide equivalent advantages if the best available first-stage chromatography operations are also less effective, which is the case for most non-mAb biologics. Precipitation effectiveness may also be improved for some products by protein engineering and/or improved screening of conditions. Otherwise, non-precipitation alternatives to chromatography—such as the use of Elastin-like polypeptides (ELPs) (Hassouneh et al., 2010), crystallization (Hubbuch et al., 2019), aqueous extraction, or others—may provide similar benefits as long as yields and selectivities are sufficiently high. Nonetheless, precipitation does have certain advantages over some of these alternatives, which may require additional steps to remove ELPs or high molecular weight polymers, or require handling of a solid-phase product after crystallization.

### Unlocking Economies of Scale in Biologic Drug Production

Our results demonstrate that replacing chromatography operations with precipitation or similar alternatives can not only decrease COGS, but shift costs to equipment and materials that experience better economies of scale. In the case of the GRFT process, COGS savings from precipitation range from ∼20% at 200 kg/yr to ∼73% at 100,000 kg/yr (Figure 1B). Liquid chromatography scales particularly poorly because increasing column height is limited by resin compression and backpressure while increasing diameter is limited by irregularities in resin packing and flow. Consequently, very large-scale processes can quickly reach a demand for column capacity that cannot be met except by using multiple columns in parallel or running each column for many cycles. In turn, facility and labor costs increase. Even for the limited amount of scale-up that is possible, resins and column hardware do not decrease in price with increasing scale as rapidly as do buffers and stirred tanks.

Previous analyses have argued that chromatography is capable of supporting production of some biologics even at the scale of 10,000 kg/yr (Kelley, 2007). Our results entirely agree with the conclusions that there are no inherent limits to throughput *per se* that keep chromatography-based biologics processes from achieving multiton production scales, or even from doing so at very low costs (Figure 1, Process A). However, the COGS for these processes nonetheless plateaus at very large scales, and some valuable applications for biologic drugs—such as a globally deployed broad-spectrum antiviral—may realistically require breaking through this plateau. One way to do so is to look beyond chromatography to operations like precipitation that are inherently capable of better economies of scale.

## Data Availability Statement

All data supporting the conclusions of this article are available within the article and its Supplementary Material. The complete bioprocess models are available upon request to the corresponding author.

## Author Contributions

JD, RM-M, and ML conceived the study and prepared the manuscript. RM-M constructed plasmids. JD and RM-M performed shake flask studies and fermentations. JD performed bioprocess modeling, design of experiments and GRFT purifications. All authors edited and revised the manuscript.

## Conflict of Interest

ML has a financial interest in DMC Biotechnologies, Inc. ML, JD, and RM-M have a financial interest in Roke Biotechnologies, Inc.
