# Supplementary material for: Low-Cost, Large-Scale Production of the Anti-viral Lectin Griffithsin
Source: Front Bioeng Biotechnol. 2020 Aug 21;8:1020. doi: 10.3389/fbioe.2020.01020 (PMC7471252; doi:10.3389/fbioe.2020.01020)
Supplement: Supplementary file 1 [file Data_Sheet_1.docx]

**Supplemental Materials**

| **Table S1: Plasmids and strain used in this study** | | | | | | |
| --- | --- | --- | --- | --- | --- | --- |
| **Plasmid** | **Insert** | **Promoter** | **Origin of Replication** | **Resistance Marker** | **Addgene #** | **Source** |
| pHC-Kan-phoAp-GRFT | GRFT | phoAp[^1^](https://paperpile.com/c/hGrzFS/zZHqf) | colE1 | Kanamycin | 158747 | This study |
| pHC-Kan-phoBp-GRFT | GRFT | phoBp[^1^](https://paperpile.com/c/hGrzFS/zZHqf) | colE1 | Kanamycin | 158746 | This study |
| pHC-Kan-yibDp-GRFT | GRFT | yibDp[^1^](https://paperpile.com/c/hGrzFS/zZHqf) | colE1 | Kanamycin | 158745 | This study |
| pHC-Kan-yibDp-Q-GRFT | GRFT | yibDp[^1^](https://paperpile.com/c/hGrzFS/zZHqf) | colE1 | Kanamycin | 158748 | This study |
| **Strains used in this study** | | | | | | |
| **Strain** | **Genotype** | | | | | **Source** |
| DLF_Z0025 | F-, λ-, Δ(araD-araB)567, lacZ4787(del)(::rrnB-3) , rph-1, Δ(rhaD-rhaB)568, hsdR514, ΔackA-pta, ΔpoxB, ΔpflB, ΔldhA, ΔadhE, ΔiclR, ΔarcA, ΔsspB::frt, Δcas3:: ugpBp-sspB-J23100p-casA | | | | | [*^2^*](https://paperpile.com/c/hGrzFS/iZ3tj) |

Section 1: *Monoclonal antibody production landscape*

In order to provide context for the potential use of biologic drugs in situations demanding large scales and low costs, such as viral pandemics, we surveyed the landscape of therapeutic monoclonal antibodies (mAbs) sold in the US in 2020. No mAb is yet known to have reached Costs of Goods Sold (COGS) below $100/g or production scales greater than 1000 kg/yr. Though mAbs are a very promising class of potential antiviral therapies, these data support the need for research into alternative biologic therapies as well as new manufacturing techniques that can reduce COGS. Sales price data reflect the lowest prices listed in 2020 procurement contracts with the US Department of Veterans Affairs, and total revenues and manufacturer COGS data are from manufacturer annual financial reports from 2019 or 2018. Drug mass sold is estimated simply by dividing total revenues by per-mass price. COGS per gram of drug is estimated by multiplying the per-gram sales price by the manufacturer’s total COGS as a fraction of total revenues. The results are shown in Figure 1A and Table S2.

Though global revenue data for each mAb should be accurate as reported by the manufacturers, the range of prices over all the sales contributing to that total is unknown, and therefore so are the estimates of drug mass sold and COGS/g. However, we have sampled prices in a way that should not give any undue support to our arguments: because we have used the lowest prices from a set of large-volume contracts with a buyer with substantial bargaining power, and because mAb sales are dominated by wealthy countries with high drug prices, this analysis should tend to approximate the true minimum price over all sales and overestimate the drug mass sold. Another uncertainty is in the actual proportion of COGS relative to sales for any particular mAb. Manufacturers only report aggregate COGS data over all their products. It is possible that any of these mAb processes actually has a significantly higher or lower ratio of COGS to sales than this company-wide average. In any case, this analysis broadly supports the trend of declining prices and COGS with increasing production scale, and suggests that it is unlikely that any mAb sold today has a true COGS far below $100/g.

| **Table S2: Market data on therapeutic mAbs sold in the US in 2020.** | | | | | |
| --- | --- | --- | --- | --- | --- |
| mAb [Manufacturer] | Lowest sales price ($/g) | Global mAb revenues ($M/yr) | Manufacturer COGS (% of total revenue) | Estimated drug mass sold (kg) | Estimated COGS ($/g) |
| Adalimumab [Abbvie] | $31,264 | $14,864 | 18.18 | 475.43 | $5,682.44 |
| Risankizumab [Abbvie] | $72,380 | $355 | 18.18 | 4.90 | $13,155.31 |
| Eculizumab [Alexion Pharmaceuticals] | $16,608 | $3,946 | 7.01 | 237.62 | $1,164.23 |
| Ravulizumab-Cwvz [Alexion Pharmaceuticals] | $16,305 | $339 | 7.01 | 20.79 | $1,142.99 |
| Erenumab-Aooe [Amgen] | $3,062 | $306 | 9.07 | 99.92 | $277.78 |
| Evolocumab [Amgen] | $1,108 | $661 | 9.07 | 596.44 | $100.52 |
| Panitumumab [Amgen] | $8,508 | $744 | 9.07 | 87.45 | $771.70 |
| Romosozumab-Aqqg [Amgen] | $6,547 | $189 | 9.07 | 28.87 | $593.88 |
| Trastuzumab-Anns [Amgen] | $5,597 | $226 | 9.07 | 40.38 | $507.66 |
| Benralizumab [Astrazeneca] | $120,425 | $686 | 16.74 | 5.70 | $20,157.08 |
| Durvalumab [Astrazeneca] | $5,242 | $1,469 | 16.74 | 280.24 | $877.42 |
| Palivizumab [Astrazeneca] | $20,849 | $269 | 16.74 | 12.90 | $3,489.69 |
| Brodalumab [Bausch Health] | $6,235 | $28 | 25.97 | 4.49 | $1,618.92 |
| Natalizumab [Biogen] | $13,119 | $1,892 | 12.33 | 144.22 | $1,618.18 |
| Elotuzumab [Bristol-Myers Squibb] | $4,615 | $357 | 25.72 | 77.35 | $1,187.09 |
| Ipilimumab [Bristol-Myers Squibb] | $108,709 | $1,489 | 25.72 | 13.70 | $27,961.44 |
| Nivolumab [Bristol-Myers Squibb] | $18,188 | $7,204 | 25.72 | 396.10 | $4,678.09 |
| Cetuximab [Eli Lilly] | $4,318 | $635 | 20.19 | 147.14 | $871.92 |
| Galcanezumab-Gnlm [Eli Lilly] | $3,561 | $163 | 20.19 | 45.63 | $719.11 |
| Ixekizumab [Eli Lilly] | $48,053 | $1,366 | 20.19 | 28.44 | $9,703.97 |
| Necitumumab [Eli Lilly] | $4,099 | $10 | 20.19 | 2.51 | $827.81 |
| Olaratumab [Eli Lilly] | $3,694 | $203 | 20.19 | 54.95 | $746.06 |
| Ramucirumab [Eli Lilly] | $8,324 | $925 | 20.19 | 111.14 | $1,680.96 |
| Avelumab [EMD Serono] | $5,814 | $79 | 21.68 | 13.58 | $1,260.69 |
| Ado-Trastuzumab Emtansine [Genentech] | $22,169 | $1,483 | 22.83 | 66.88 | $5,061.62 |
| Atezolizumab [Genentech] | $5,553 | $1,996 | 22.83 | 359.37 | $1,267.86 |
| Bevacizumab [Genentech] | $5,483 | $7,528 | 22.83 | 1372.97 | $1,251.87 |
| Emicizumab-Kxwh [Genentech] | $62,444 | $1,424 | 22.83 | 22.80 | $14,257.47 |
| Obinutuzumab [Genentech] | $4,646 | $587 | 22.83 | 126.43 | $1,060.81 |
| Ocrelizumab [Genentech] | $40,740 | $3,946 | 22.83 | 96.85 | $9,301.98 |
| Omalizumab [Genentech] | $5,256 | $2,095 | 22.83 | 398.62 | $1,200.17 |
| Pertuzumab [Genentech] | $8,632 | $3,748 | 22.83 | 434.21 | $1,970.96 |
| Polatuzumab Vedotin-Piiq [Genentech] | $81,838 | $54 | 22.83 | 0.66 | $18,685.59 |
| Ranibizumab [Genentech] | $1,715,533 | $1,943 | 22.83 | 1.13 | $391,698.64 |
| Rituximab [Genentech] | $6,089 | $6,892 | 22.83 | 1131.88 | $1,390.28 |
| Tocilizumab [Genentech] | $3,478 | $2,459 | 22.83 | 707.09 | $794.06 |
| Trastuzumab [Genentech] | $7,402 | $6,426 | 22.83 | 868.17 | $1,690.00 |
| Alemtuzumab [Genzyme] | $1,361,914 | $293 | 28.76 | 0.22 | $391,621.47 |
| Caplacizumab-Yhdp [Genzyme] | $494,383 | $64 | 28.76 | 0.13 | $142,160.86 |
| Belimumab [GlaxoSmithKline] | $3,152 | $775 | 25.66 | 246.00 | $808.92 |
| Mepolizumab [GlaxoSmithKline] | $21,260 | $972 | 25.66 | 45.70 | $5,455.68 |
| Daratumumab [Johnson & Johnson] | $3,135 | $2,998 | 24.45 | 956.18 | $766.66 |
| Golimumab [Johnson & Johnson] | $17,556 | $2,188 | 24.45 | 124.63 | $4,292.66 |
| Guselkumab [Johnson & Johnson] | $74,264 | $1,012 | 24.45 | 13.63 | $18,158.89 |
| Infliximab [Johnson & Johnson] | $5,451 | $4,380 | 24.45 | 803.60 | $1,332.74 |
| Ustekinumab [Johnson & Johnson] | $8,876 | $6,361 | 24.45 | 716.63 | $2,170.41 |
| Pembrolizumab [Merck] | $34,580 | $11,084 | 21.68 | 320.54 | $7,498.19 |
| Canakinumab [Novartis] | $79,994 | $671 | 24.29 | 8.39 | $19,431.31 |
| Secukinumab [Novartis] | $21,945 | $3,551 | 24.29 | 161.82 | $5,330.53 |
| Cemiplimab-Rwic [Regeneron] | $19,769 | $176 | 59.64 | 8.90 | $11,789.98 |
| Alirocumab [Sanofi Aventis] | $1,462 | $271 | 28.76 | 185.35 | $420.49 |
| Dupilumab [Sanofi Aventis] | $7,195 | $2,339 | 28.76 | 325.09 | $2,068.88 |
| Sarilumab [Sanofi Aventis] | $4,844 | $209 | 28.76 | 43.20 | $1,392.83 |
| Brentuximab Vedotin [Seattle Genetics] | $108,120 | $628 | 6.75 | 5.81 | $7,292.97 |
| Fremanezumab-Vfrm [Teva Pharmaceuticals] | $1,909 | $93 | 47.09 | 48.72 | $898.88 |
| Dinutuximab [United Therapeutics] | $377,992 | $114 | 10.00 | 0.30 | $37,803.84 |

Section 2: *Plant-based GRFT production*

To estimate the potential cost of goods sold (COGS) for plant-based routes to cGMP GRFT at various production scales, we analyzed the COGS structure of a process model previously published by Alam *et al.*, which was designed to produce 20 kg per year of GRFT API.[^3^](https://paperpile.com/c/hGrzFS/wbp9) Specifically, we applied commonly-used exponential economy of scale heuristics (see Equation 1) to achieve order-of-magnitude estimates after adapting the cost structure of Alam *et al.*’s model to reflect a few key differences associated with larger-scale production.

**Equation 1**: $P_{LS}=P_{ss}*(\frac{LS}{ss})^{k}$. An exponential economy of scale model widely used to estimate prices in bioprocessing and other industries.[^4,5^](https://paperpile.com/c/hGrzFS/Jef5+909J) LS and ss are the large and small scales of consumption of some good, respectively; P denotes price; k is the exponential scaling factor.

Because the relatively small-scale model of Alam *et al.* assumes production by a contract manufacturing organization, it includes no facility-related costs except equipment maintenance. However, similar processes capable of producing thousands of kg of GRFT per year from tobacco would require construction of dedicated manufacturing plants and would thus incur additional facility-related operating costs such as equipment depreciation, insurance, and taxes. Therefore, we extrapolated from the maintenance costs reported by Alam *et al.* by assuming that they correspond to 6% of total fixed capital costs for the plant, as in our model and as is the default for SuperPro Designer. We then calculated depreciation, insurance, taxes, and miscellaneous expenses from this fixed capital cost, again in the same way as was done for our models and using SuperPro’s default options. Specifically, we assumed 10-year, straight-line depreciation of all fixed capital down to 5% of the initial value, as well as taxes, insurance, and miscellaneous expenses corresponding to 2%, 1%, and 5% of the fixed capital costs, respectively. Table S3, adapted from Table 2 of Alam *et al.*’s original publication, shows their model’s COGS structure updated to reflect the aforementioned extrapolations. For comparison, the COGS structure of the 24,000 kg per year, precipitation-based *E. coli* bioprocess model (main text figure 2, Process B) is shown in Table S4.

| **Table S3:** Cost breakdown of a tobacco-based process designed to produce 20 kg per year of cGMP GRFT API. Adapted from [[^3^](https://paperpile.com/c/hGrzFS/wbp9)]. Extrapolations from the published model are marked by an asterisk and described in the text. | | |
| --- | --- | --- |
| Cost Category | COGS per kg GRFT API | % of total |
| Materials (i.e., chemicals, water, etc.) | $7,659 | 5.58 |
| Equipment maintenance | $10,573 | 7.70 |
| *Equipment depreciation | $16,740 | 12.19 |
| *Facility-related insurance, taxes, and miscellaneous expenses | $14,097 | 10.27 |
| Labor | $32,893 | 23.96 |
| Lab/QC | $5,086 | 3.70 |
| Consumables (e.g., resins, membranes) | $12,796 | 9.32 |
| Utilities | $36,643 | 26.69 |
| Waste treatment | $792 | 0.58 |
| Total | $137,279 | 100 |

| **Table S4:** Cost breakdown of an *E. coli* fermentation-based process using a precipitation-based purification scheme, designed to produce 24,000 kg per year of formulated and filled GRFT antiviral. See main text Figure 2, Process B. | | |
| --- | --- | --- |
| Cost Category | COGS per kg GRFT | % of total |
| Materials (i.e., chemicals, water, etc.) | $1,399.98 | 42.69 |
| Facility-related operating costs | $990.65 | 30.21 |
| Labor and QC | $639.51 | 19.50 |
| Consumables (i.e., resins and membranes) | $227.75 | 6.94 |
| Other (waste treatment and utilities) | $21.44 | 0.65 |
| Total | $3,279.33 | 100 |

Comparing these two cost structures, they are similar in the percentages of COGS allocated to facility costs (both approximately 30%), labor and QC (28% vs. 20%), and consumables (9% vs. 7%). However, in Alam *et al.*’s model, the vast majority of remaining COGS are made up of utilities costs associated with growing plant biomass, while in the precipitation-based *E. coli* model they are made up almost entirely of raw materials. This difference is highly significant when considering likely economies of scale behavior: commodity chemicals tend to enjoy fairly strong economies of scale (exponential scaling factors of approximately -0.4 to -0.7)[^5^](https://paperpile.com/c/hGrzFS/909J); in contrast, electricity rates do not typically reflect usage discounts beyond broad categories such as residential vs. industrial, and in many cases higher usage even results in increased rates within these categories due to the stress placed on power grids.

Finally, we estimate COGS for large-scale tobacco-based GRFT production by applying exponential scaling to the costs detailed in Table S3. We assume a common rule-of-thumb scaling factor of -0.6 for all bioprocessing equipment.[^4^](https://paperpile.com/c/hGrzFS/Jef5) However, true economies of scale in this case may be more modest because of the less efficient use of space involved with growing plant biomass versus bacterial biomass, leading to dramatically larger building and equipment sizes with increasing production scale. We also assume a factor of -0.4 for raw materials (chemicals and water for injection), as used for our models (see Section 3) and broadly consistent with other published values.[^5^](https://paperpile.com/c/hGrzFS/909J) Because employee salaries do not decline with the number of employees, we assume a factor of 0 for labor and QC. We also assume that discounts for consumables will be minimal once the production scale exceeds hundreds of kg, as very few biologic drug processes operate at capacities in excess of this; we therefore assign a scaling factor of 0, as was also used in our models. Finally, we assume factors of 0 for waste treatment and for utilities, for the reasons previously described. The results of this analysis are shown in Table S5, which also includes estimates of the amount of tobacco needed to produce various amounts of GRFT, based on the yields given by Alam *et al.*

| **Table S5:** Estimated COGS per kg GRFT API produced in tobacco, by production scale and cost category. These estimates are intended to be illustrative, but are expected to provide order-of-magnitude accuracy only. | | | | |
| --- | --- | --- | --- | --- |
|  | | Annual production capacity | | |
|  |  |  |  |  |
| **Cost category** | **Scaling factor (k)** | **200 kg/yr** | **2,000 kg/yr** | **20,000 kg/yr** |
| Materials | -0.4 | $3,049.02 | $1,213.84 | $483.24 |
| Consumables | 0 | $12,796.10 | $12,796.10 | $12,796.10 |
| Facility | -0.6 | $10,401.46 | $2,612.73 | $656.29 |
| Labor/QC | 0 | $37,978.35 | $37,978.35 | $37,978.35 |
| Utilities | 0 | $36,642.95 | $36,642.95 | $36,642.95 |
| Waste treatment | 0 | $791.55 | $791.55 | $791.55 |
| **Total COGS per kg GRFT API** | | $101,659.43 | $92,035.52 | $89,348.48 |
| **Total tobacco required (metric tons)** | | 385 | 3,846 | 38,462 |
| **Percentage of total 2018 US tobacco crop required.**[**^6^**](https://paperpile.com/c/hGrzFS/qifj) | | 0.16% | 1.59% | 15.90% |

The cost estimation analysis presented here is admittedly and necessarily naïve: the authors are not aware of any examples of plant-based biologic drug processes operating at the scale of hundreds or thousands of kg of product per year, either in existing processes or detailed models. There is therefore a large degree of uncertainty in how such processes would scale and how their COGS would be structured. Likewise, we are not aware of published data or common heuristics related to economies of scale for specialty biotechnology consumables, waste treatment, or very large-scale electricity usage. Furthermore, labor costs may experience some economies of scale not reflected here, both because of increased labor efficiency in larger plants and because of lower rates for indirect costs such as insurance and healthcare. However, we believe that these estimates nonetheless provide order-of-magnitude accuracy and suffice to show that tobacco-based routes to GRFT antivirals are unlikely to be cost-competitive with *E. coli*-based processes at large scales. To check our assumptions, we applied the same analysis to a scale-down of our 20,000 kg GRFT per year, precipitation-based model (main text figure 2B, Process B) and compared the cost estimates for various production scales produced by this naïve approach to those produced by detailed SuperPro models. Compared to the detailed models, the naïve approach based on exponential scaling underestimated COGS at the 2,000 and 200 kg per year scales by 0.33 and 0.82 log_10_ units, respectively. Thus, while the exponential scaling approach is far from exact, it seems to be capable of maintaining order-of-magnitude accuracy in COGS estimates over more than two orders of magnitude in bioprocess scale. Furthermore, for the tobacco-based process to be competitive with the *E. coli* process (Process B) at a 20,000 kg GRFT per year scale, labor/QC and utilities costs per kg GRFT would each have to fall by a factor of approximately 20 from their values at the 20 kg GRFT per year scale, which seems highly unlikely. Finally, tobacco-based production of tens of thousands of kgs of GRFT per year, as might be required for applications such as pandemic antiviral treatment or HIV microbicides, would be expected to require a very significant fraction of the total US annual tobacco crop. For all these reasons, it seems unlikely that plant-based routes to GRFT can meet the demands of these large-scale applications.

Section 3: *E. coli* *Fermentation-Based GRFT Bioprocess Models*

*General Design Considerations*

Here we present variations on a batch bioprocess design for GRFT production by *E. coli* fermentation. Each process is designed such that batch throughput and cycle time are limited by the production fermentation procedure and other equipment is sized to meet the consequent material or throughput demands without excess capacity. Maximum feasible equipment capacities or throughputs were set according to default SuperPro options, except where noted. To account for processing delays and operations not included in the model, a minimum of 4 hours is allowed between operations for consecutive batches in each piece of equipment. The plant is assumed to be operational for 85% of each year. Each process comprises five main sections: seed train, fermentation, primary recovery, purification, and formulation. Additionally, we modeled two different purification scenarios, one based on the optimized precipitation step described in this report, and another based on a more typical approach using three chromatography columns. We then conducted sensitivity analyses and scaling analyses on both models, as described below.

*Auxiliary Operations*

All non-disposable equipment, except chromatography columns, undergoes CIP and SIP operations once per batch. CIP cycles are assumed to take 110 minutes and include two rinses with potable water, one rinse each with 5% v/v H_3_PO_4_ and 0.5 M NaOH, and a final rinse with WFI. SIP cycles are assumed to take 50 minutes with 30 minutes of steaming. Columns are sanitized with 0.5 M NaOH for 4 hr. TFF skids are assumed to require flushing and equilibration with 20 L/m^2^ of WFI and buffer, respectively. Buffer preparation operations are explicitly modeled by including stainless steel blending tanks of the necessary sizes and allocating 1 hour of labor each for preparation and validation, in addition to labor for the duration of material transport to buffer-consuming unit operations. Columns, TFF skids, and buffer preparations are each also assumed to require 1 hour per cycle for testing and validation. The cost of CIP skids, but not SIP panels, is included according to built-in cost models based on the volumes of cleaning solutions required. All waste disposal costs are assumed to be $0.01/kg waste. For simplicity, some other auxiliary operations and equipment categories are not explicitly modeled, including materials storage for in-process pools or raw materials, packaging and storage of finished goods, and activities not directly related to goods production.

*Economic Considerations*

Because we are specifically interested in the potential of GRFT-based antivirals for epidemic response, we consider only large-scale production (20000+ kg GRFT/yr) with a requirement for relatively low costs. We assume that these considerations will make the use of contract manufacturing infeasible and necessitate the construction of a single-product plant from greenfield. The plant is financed by a 60:40 mix of debt and equity with loans amortized over 10 years at 9% interest, and all capital cost items are subject to straight-line depreciation over 10 years. Except where noted, SuperPro’s default built-in cost models were used to estimate expenses for equipment purchase, other capital investment including unlisted equipment and other facility improvements, insurance and taxes, maintenance, utilities and consumables. Labor demand was estimated by assigning a value of 1 labor hour per operating hour to the large majority of operations, with the exception of prolonged, highly automated processes such as monitoring agitation in stirred tanks (0.1 labor hours per operating hour) and operations involving material transfer between two pieces of equipment (2 labor hours per operating hour, to account for labor at the source and destination operations). The total expense rate for plant operators, including benefits and overhead, was estimated as $80.50/hr. Quality control costs were estimated as 25% of all manufacturing-related labor costs. Chemicals, final product vials, and WFI were priced according to the amount consumed annually per process using an exponential economies of scale model. Baseline prices and scales were obtained from vendor quotes or from the literature, and a scaling exponent of -0.4 was chosen because it provided the best fit between small- and large-scale quotes where both were available.

*Seed Train*

300 L of inoculum are needed per batch to support production-scale fermentation. In the present models we omit primary and secondary seed cultures (e.g., in shake flasks and roller bottles) and begin with a 300 L culture in a stainless steel seed fermenter. Seed fermenter media is prepared in a separate stainless steel tank. Media sterilization operations (typically dead-end filtration) are also omitted. The seed culture is allowed to grow for 24 hours. Because clean in place (CIP) and steam in place (SIP) operations are required for the seed fermenter and cause its cycle time to slightly exceed the batch cycle time, two seed tanks are run in staggered mode to avoid cycle time increases. Seed train operations require a total of approximately 32 hours in each batch.

*Fermentation*

Production-scale fermentation takes place in two stainless steel tanks each holding a working volume of 30000 L and operating on a staggered schedule. Media for these tanks is prepared in a separate stainless steel blending tank. We assume a two-stage fermentation in which the culture grows to a density of 100 grams dry cell weight per L (gDCW/L) within 24 hours and protein is expressed during stationary phase for 24 hours. The yield of biomass from glucose is assumed to be 50%. With 48 hours of fermentation time in addition to CIP/SIP and material transfer operations, the fermentation procedure is the longest in the batch at 56 hours. Thus, with two fermenters operating on a staggered schedule, the minimum process cycle time is 28 hours in all models. All other unit procedures are designed so that their procedure cycle times do not exceed this limit, to avoid lengthening the process cycle time. Fermentation media preparation begins with 25 hours elapsed in the batch, and the section ends with the conclusion of tank CIP/SIP operations at approximately 83 hours elapsed.

*Primary Recovery*

After each fermentation, the 30000 L culture is harvested by disk-stack centrifugation at a flow rate of 1500 L/h. It is assumed that all biomass is recovered in a volume of approximately 15000 L. The cell slurry is then diluted back to 30000 L by the addition of lysis buffer (PBS) before high-pressure homogenization. Homogenization is achieved by 3 passes with a pressure drop of 700 bar and a flow rate of 4000 L/h; it is assumed that 99% of cell contents are released and protein denaturation is ignored. The composition of cell dry weight is assumed to be 5% GRFT, 45% host cell proteins (HCPs), 3.1% DNA, 20.5% RNA, 3.4% lipopolysaccharide (LPS), and 23% other insoluble debris.[^7^](https://paperpile.com/c/hGrzFS/D1iP) Primary recovery continues with another centrifugation step to remove insoluble cell debris, again at a flow rate of 1500 L/h. We assume that all debris is removed, with negligible volume change or loss of other cell components. Cell harvest begins with 77 hours elapsed in each batch, and CIP/SIP operations in the lysate clarification centrifuge conclude at 142 hours elapsed.

*Purification*

The purification section is designed to produce GRFT bulk drug substance (BDS) with the following purities relative to three major categories of contaminants: for HCPs, <100 ppm[^8^](https://paperpile.com/c/hGrzFS/o3oV); for nucleic acids, <10 ng per dose[^9^](https://paperpile.com/c/hGrzFS/jVdS), i.e. <100 pg/mg GRFT assuming a maximum dose of 100 mg; and for LPS, <1.25 ng/mg GRFT, assuming a limit of 5 EU/kg patient bodyweight, a dose of 0.4 mg GRFT/kg, and a mass of 0.1 ng LPS per EU. Considering the cell composition given above, the total-process minimum separation factors (ɑ) needed for HCPs, nucleic acids, and LPS are 9*10^4^, 6.18*10^6^, and 5.44*10^5^, respectively. We modeled two different purification scenarios to achieve these targets: a process based on a precipitation step followed by one diafiltration step and two chromatography steps; and a conventional process based on three chromatography steps and two diafiltration steps.

For all diafiltration steps in all models, we assume tangential flow filtration (TFF) with a filtrate flux of 30 L/m^2^-h, a retention coefficient of 0 for buffer salts and 1 for all macromolecules, constant-volume operation, membrane replacement every 1000 operating hours, and a maximum membrane area per skid of 100 m^2^. For all chromatography steps, we assume a fixed bed height of 250 cm, a maximum column diameter of 2 m, a constant flow rate of 150 cm/hr and resin replacement every 100 cycles. For anion and cation exchange columns operated in bind-and-elute mode, we assume a total loading capacity of 100 g/L and 120 g/L, respectively. Both kinds of columns are sized to accommodate the initial interaction with the resin of 100% of each of the four macromolecular species (GRFT, HCPs, nucleic acids, and LPS) present in the feed. We assume that all bind-and-elute chromatography procedures include equilibration with 6 BVs of loading buffer, loading, washing with 5 bed volumes (BVs) of loading buffer, elution with 4 BVs of elution buffer, and regeneration with 4 BVs of regeneration buffer. For flow-through chromatography, we omit the wash and elution steps and size the column to accommodate the bound species that demands the highest capacity (i.e., that has the highest value of mass in feed divided by species-specific loading capacity). Each chromatography step is assumed to have 90% yield.

The precipitation-based purification process begins with a 2.5-fold dilution of the clarified cell lysate from 50 g/L total protein to the precipitation working concentration of 20 g/L in a solution containing (NH_4_)_2_SO_4_ and strong acid. The result is approximately 75000 L of lysate per batch at pH 3.4 and an (NH_4_)_2_SO_4_ concentration of 0.82 M (20% saturated at 25 °C). The diluted lysate is transferred to an 85000 L stainless steel tank and heated to 60 °C over 30 minutes, then held at 60 °C and stirred for an additional 30 minutes. During this time contaminants are precipitated with the following separation factors, as demonstrated at lab scale for our optimized precipitation step: 426 for HCPs (Figure 4), 1000 for LPS (as measured by Pierce™ Chromogenic Endotoxin Quant Kit; Thermo Fisher Scientific, Waltham, MA), and 14400 for DNA (Supplementary Figure S1). To meet final purity targets, the remaining purification steps in this scenario must therefore provide additional separation factors of >211, >544, and >429.2 for HCPs, LPS and DNA, respectively.

Following the precipitation treatment, precipitate clearance and buffer exchange are required. The suspension is first transferred to a disk-stack centrifuge operating at a flow rate of 3000 L/h. We assume 100% removal of precipitates, with a consequent reduction in volume to about 71000 L. The process stream is then transferred to a TFF procedure for diafiltration and concentration. To minimize the buffer volume used in diafiltration and loading for the final chromatography step, as well as the membrane area needed for diafiltration, the feed is first concentrated to a total protein concentration of approximately 20 g/L (10.8-fold concentration in the baseline model). The approximately 6750 L of concentrated feed are then diafiltered into citrate buffer for loading on a cation exchange column. Reducing the (NH_4_)_2_SO_4_ concentration from the 0.82 M used in the precipitation step to a level suitable for loading on a cation exchange column (assumed to be 15 mM) requires processing 4 diafiltration volumes. Given the general TFF assumptions stated above, completing these steps without an increase in the batch cycle time requires two TFF skids running in parallel with 70 m^2^ of membrane each.

After TFF, the feed stream is loaded onto a cation exchange column in bind-and-elute mode. The total load of GRFT and contaminants can be accommodated with approximately 574 L of resin, given that the column can be cycled twice. Ion exchange steps typically provide 1-2 log clearance of HCPs[^10,11^](https://paperpile.com/c/hGrzFS/VeNs+sDG2Q), and the significant majority of *E. coli* HCPs are more basic than GRFT. Likewise, the strong negative charge and low protein-binding of DNA are known to permit 3-log clearances from proteins that bind to cation exchange columns, and LPS should behave similarly albeit perhaps with more binding to protein. Therefore this step is assumed to provide separation factors of 1000, 2000, and 50 for LPS, DNA, and HCPs, respectively.

After elution from the cation exchange column, the eluate is loaded directly into a flow-through strong anion exchange chromatography step. We follow the method of Chen *et al.*[*^12^*](https://paperpile.com/c/hGrzFS/JI7VS), in which a pH between the isoelectric points of GRFT (5.4) and LPS (approximately 2) causes both LPS and nucleic acids to be attracted to the resin far more strongly than are proteins, while 50 mM (NH_4_)_2_SO_4_ is used to screen charge attractions between proteins and these other contaminants. In our hands, and consistent with the findings of Chen *et al.*, a version of this procedure at lab scale provided an ɑ for LPS of approximately 1000 (data not shown). Because the charge attraction of nucleic acids to the resin should be even greater than for LPS, we assume this step achieves an ɑ of 2000 for DNA and 1000 for LPS. We also assume an ɑ of 50 for HCPs on the same basis as for the cation exchange step. We assume a loading capacity of 0.09 g/L for LPS[^12^](https://paperpile.com/c/hGrzFS/JI7VS) and 6–12 g/L for nucleic acids[^13^](https://paperpile.com/c/hGrzFS/zcrNo). The required resin volume for this column, again operated in two cycles, is only 62 L. We assume that GRFT can be eluted from the cation exchange column in 50 mM (NH_4_)_2_SO_4_ so that no intermediate diafiltration steps are required.

The precipitation-based purification scenario ends with a total of 4800 L of GRFT BDS per batch collected from the column, containing 22.5 g GRFT/L, 0.19 mg HCPs/L (8.44 ppm), ~15.3 pg LPS/L, and ~0.485 pg DNA/L (< 0.0003 ng/mg GRFT). From the beginning of lysate dilution in precipitation buffer at 140 hours elapsed to the end of operations in the flow-through chromatography procedure at 215 hours elapsed, the precipitation-based purification section requires a total of 75 hours. The overall yield of the precipitation-based process is 74.1%.

The conventional purification process begins with TFF diafiltration of the clarified cell lysate from lysis buffer into cation exchange loading buffer (citrate). We assume that 95% buffer exchange (3 diafiltration volumes) is sufficient for this step. Completing this step without an increase in the batch cycle time requires two TFF skids with 70 m^2^ of membrane each. Next, the filtrate is loaded onto a strong cation exchange column running in bind-and-elute mode. The procedure is expected to operate as previously described under the precipitation scenario, except that the high contaminant load requires much more resin volume. Specifically, this operation requires 6940 L of resin spread across ten columns running in parallel, each cycled twice.

The eluate from the first cation exchange step is then loaded directly onto a flow-through anion exchange column, also as described under the precipitation scenario. After passing through the column, the GRFT fraction is passed to another 80 m^2^ TFF skid to be concentrated to a total protein concentration of approximately 20 g/L and diafiltered with 4 diafiltration volumes. The final step in the conventional purification process is bind-and-elute strong anion exchange chromatography, to complete removal of acidic HCPs. We assume separation factors for this step of 10, 10, and 36 for LPS, DNA and HCPs, respectively.

The conventional purification scenario produces 5000 L of GRFT BDS per batch, containing 21.3 g GRFT/L, 0.26 μg DNA/L (12.21 pg/mg GRFT), 2.1 mg HCPs/L (100 ppm), and 1.45 μg LPS/L (0.681 ng/mg GRFT). The conventional purification process begins with 142 hours elapsed in each batch and concludes with 229 hours elapsed. The overall yield of the conventional process is 72.9%.

*Formulation and Finishing*

Following collection of GRFT BDS at the end of each purification scenario, formulation begins with concentration of the BDS and its diafiltration into PBS. We assume concentration to ~20 g GRFT/L and 98% buffer exchange accomplished in 4 diafiltration volumes. This requires a TFF skid with 30 m^2^ of membrane. After diafiltration, excipients are added. Following from typical formulations used for nebulization of proteins,[^14,15^](https://paperpile.com/c/hGrzFS/cECm+wmEA) we assume a final formulation of 20 g GRFT/L with 8% w/w hydroxyethyl starch and 0.1% w/w polysorbate 80. The product is finished by aseptic filling of 10 mL (200 mg) multi-dose vials. At the time of writing, the authors are aware of suitable filling machines with throughputs of up to 24000 units per hour. In the absence of detailed cost data, we assume a baseline purchase cost of $3000000 for a machine with 12000 unit/hr throughput (from SuperPro documentation) and a throughput-based cost model with a scaling exponent of 0.6. The final product streams are approximately 544766 or 53583 filled vials per batch for the precipitation-based and conventional purification scenarios, respectively. Formulation and finishing operations run from 221 to 269 hours elapsed in the conventional purification scenario or 209 to 257 hours elapsed in the precipitation-based scenario.

**Supplemental Figures**

**
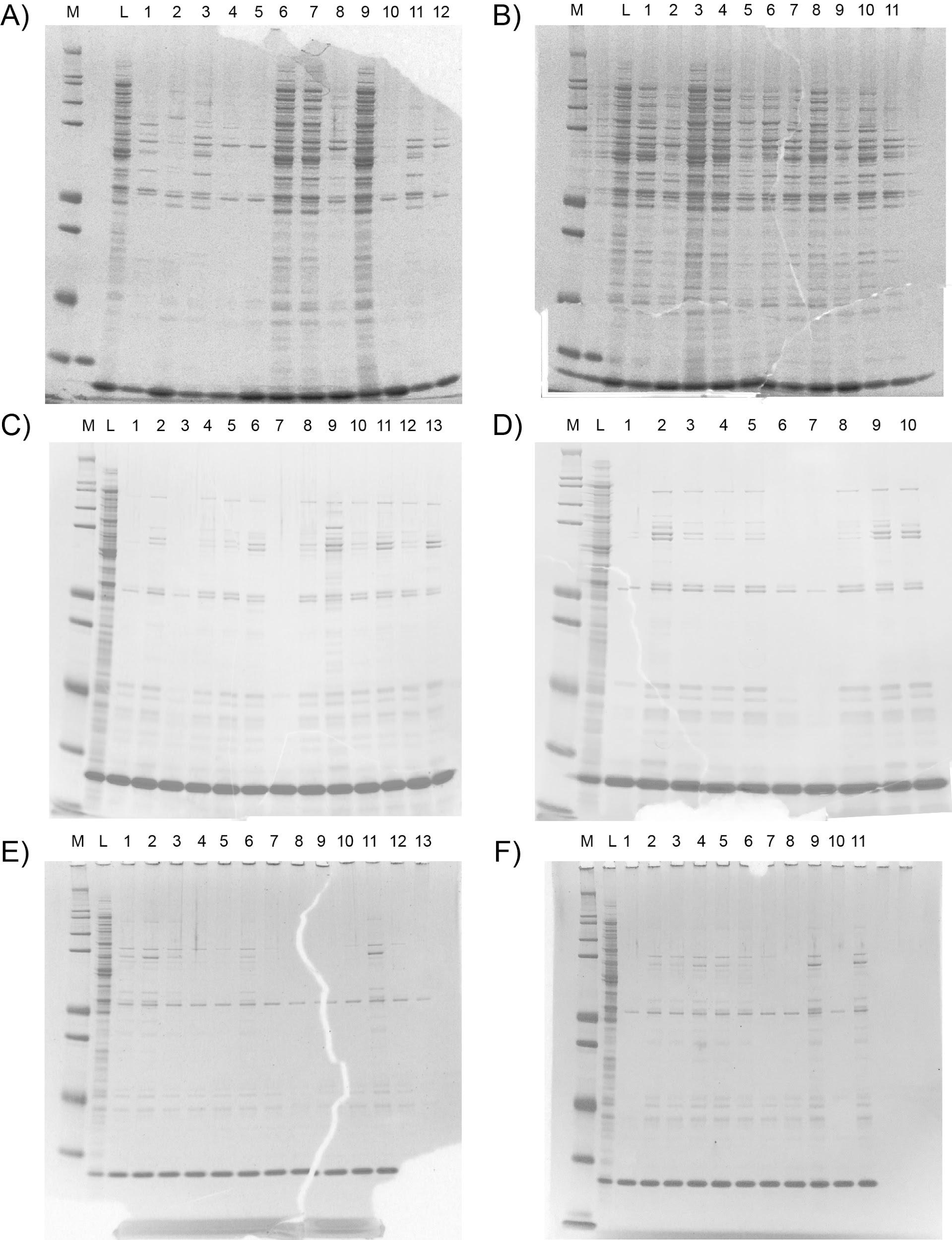
**

**Supplementary Figure S1: Optimization of GRFT precipitation**. A-F: SYPRO Ruby-stained SDS-PAGE gels showing supernatants remaining after precipitation of contaminants, converted to grayscale and inverted for clarity. A-D, numbers are for labeling purposes only. E and F, numbers represent total protein concentrations at the start of the precipitation step. M: molecular weight marker. L: untreated lysate standard (see Materials and Methods). A, B: initial definitive screening round. C, D: second optimization round. The optimal condition was sample 7 in D, initially found with 2.5 g total protein/L entering the precipitation step. Modeling suggested 20 g/L as necessary to support cost targets. E, F: validation of precipitation step with high purity and yield up to 20 g/L.Refer to Supplemental Table S6 for gel details and processed results.

| **Supplementary Table S6** | | | | | | | | |
| --- | --- | --- | --- | --- | --- | --- | --- | --- |
| Gel | Sample Label | Temp (° C) | (NH_4_)_2_SO_4_ (% saturation) | pH | Time (min) | Protein conc. (g/L) | % Yield | Separation Factor |
| A | 1 | 37.0 | 50 | 4.4 | 5 | 5.00 | 26.65 | 3.43 |
| A | 2 | 65.0 | 20 | 10.0 | 60 | 5.00 | 51.46 | 10.76 |
| A | 3 | 51.0 | 50 | 10.0 | 60 | 5.00 | 26.71 | 2.42 |
| A | 4 | 65.0 | 50 | 4.4 | 60 | 5.00 | 37.46 | 14.82 |
| A | 5 | 51.0 | 20 | 4.4 | 5 | 5.00 | 57.84 | 21.56 |
| A | 6 | 37.0 | 35 | 10.0 | 60 | 5.00 | 57.47 | 0.73 |
| A | 7 | 51.0 | 35 | 7.2 | 32.5 | 5.00 | 51.34 | 1.23 |
| A | 8 | 65.0 | 35 | 4.4 | 5 | 5.00 | 56.00 | 5.60 |
| A | 9 | 37.0 | 20 | 10.0 | 5 | 5.00 | 48.98 | 0.59 |
| A | 10 | 65.0 | 20 | 4.4 | 32.5 | 5.00 | 58.91 | 35.38 |
| A | 11 | 65.0 | 50 | 10.0 | 5 | 5.00 | 33.99 | 4.40 |
| A | 12 | 65.0 | 50 | 4.4 | 60 | 5.00 | 42.00 | 15.01 |
| B | 1 | 37.0 | 50 | 10.0 | 32.5 | 5.00 | 47.16 | 1.16 |
| B | 2 | 37.0 | 20 | 4.4 | 60 | 5.00 | 64.46 | 3.77 |
| B | 3 | 37.0 | 20 | 10.0 | 5 | 5.00 | 62.27 | 0.68 |
| B | 4 | 51.0 | 35 | 7.2 | 32.5 | 5.00 | 65.41 | 1.34 |
| B | 5 | 65.0 | 20 | 10.0 | 60 | 5.00 | 60.71 | 2.26 |
| B | 6 | 65.0 | 20 | 7.2 | 5 | 5.00 | 42.44 | 1.78 |
| B | 7 | 37.0 | 50 | 4.4 | 5 | 5.00 | 54.56 | 1.84 |
| B | 8 | 51.0 | 35 | 7.2 | 32.5 | 5.00 | 82.21 | 1.18 |
| B | 9 | 37.0 | 20 | 4.4 | 60 | 5.00 | 70.02 | 2.75 |
| B | 10 | 37.0 | 50 | 7.2 | 60 | 5.00 | 39.89 | 1.02 |
| B | 11 | 65.0 | 50 | 10.0 | 5 | 5.00 | 36.42 | 1.03 |
| C | 1 | 73.3 | 20 | 4.4 | 60 | 5.00 | 81.49 | 57.64 |
| C | 2 | 65.0 | 20 | 4.4 | 60 | 5.00 | 98.30 | 16.88 |
| C | 3 | 60.0 | 20 | 3.4 | 60 | 7.50 | 88.55 | 148.67 |
| C | 4 | 65.0 | 20 | 4.4 | 60 | 5.00 | 78.82 | 33.15 |
| C | 5 | 65.0 | 20 | 4.4 | 60 | 5.00 | 89.10 | 21.44 |
| C | 6 | 56.7 | 20 | 4.4 | 60 | 5.00 | 89.23 | 21.15 |
| C | 7 | 65.0 | 20 | 2.7 | 60 | 5.00 | 103.20 | 252.29 |
| C | 8 | 65.0 | 20 | 4.4 | 60 | 5.00 | 99.62 | 20.08 |
| C | 9 | 65.0 | 20 | 6.1 | 60 | 5.00 | 90.32 | 7.12 |
| C | 10 | 65.0 | 20 | 4.4 | 60 | 5.00 | 97.18 | 20.37 |
| C | 11 | 65.0 | 20 | 4.4 | 60 | 9.17 | 89.96 | 12.71 |
| C | 12 | 65.0 | 20 | 4.4 | 60 | 5.00 | 92.84 | 23.69 |
| C | 13 | 60.0 | 20 | 5.4 | 60 | 7.50 | 86.92 | 15.37 |
| D | 1 | 70.0 | 20 | 3.4 | 60 | 2.50 | 81.46 | 68.70 |
| D | 2 | 70.0 | 20 | 5.4 | 60 | 2.50 | 104.75 | 10.57 |
| D | 3 | 65.0 | 20 | 4.4 | 60 | 5.00 | 95.95 | 14.64 |
| D | 4 | 65.0 | 20 | 4.4 | 60 | 5.00 | 99.91 | 31.26 |
| D | 5 | 65.0 | 20 | 4.4 | 60 | 5.00 | 92.95 | 20.21 |
| D | 6 | 70.0 | 20 | 3.4 | 60 | 7.50 | 91.34 | 94.46 |
| D | 7 | 60.0 | 20 | 3.4 | 60 | 2.50 | 91.48 | 425.65 |
| D | 8 | 65.0 | 20 | 4.4 | 60 | 0.83 | 109.55 | 23.54 |
| D | 9 | 70.0 | 20 | 5.4 | 60 | 7.50 | 89.16 | 13.25 |
| D | 10 | 60.0 | 20 | 5.4 | 60 | 2.50 | 88.92 | 11.34 |
| E | 1 | 60.0 | 20 | 3.4 | 5 | 35.00 | 101.66 | 30.06 |
| E | 2 | 60.0 | 20 | 3.4 | 10 | 50.00 | 111.18 | 16.66 |
| E | 3 | 60.0 | 20 | 3.4 | 20 | 50.00 | 96.00 | 34.23 |
| E | 4 | 60.0 | 20 | 3.4 | 5 | 20.00 | 117.26 | 58.26 |
| E | 5 | 60.0 | 20 | 3.4 | 10 | 20.00 | 123.97 | 86.99 |
| E | 6 | 60.0 | 20 | 3.4 | 10 | 35.00 | 118.79 | 32.44 |
| E | 7 | 60.0 | 20 | 3.4 | 20 | 20.00 | 124.12 | 103.99 |
| E | 8 | 60.0 | 20 | 3.4 | 20 | 5.00 | 149.47 | 240.29 |
| E | 9 | 60.0 | 20 | 3.4 | 5 | 5.00 | 142.64 | 231.71 |
| E | 10 | 60.0 | 20 | 3.4 | 5 | 20.00 | 147.02 | 115.73 |
| E | 11 | 60.0 | 20 | 3.4 | 20 | 50.00 | 149.25 | 24.46 |
| E | 12 | 60.0 | 20 | 3.4 | 10 | 20.00 | Not analyzed | #N/A |
| E | 13 | 60.0 | 20 | 3.4 | 10 | 5.00 | Not analyzed | #N/A |
| F | 1 | 60.0 | 20 | 3.4 | 20 | 5.00 | 87.11 | 226.88 |
| F | 2 | 60.0 | 20 | 3.4 | 10 | 35.00 | 89.98 | 33.16 |
| F | 3 | 60.0 | 20 | 3.4 | 20 | 35.00 | 89.74 | 46.29 |
| F | 4 | 60.0 | 20 | 3.4 | 5 | 35.00 | 95.47 | 21.92 |
| F | 5 | 60.0 | 20 | 3.4 | 5 | 50.00 | 101.85 | 26.78 |
| F | 6 | 60.0 | 20 | 3.4 | 20 | 35.00 | 89.81 | 37.10 |
| F | 7 | 60.0 | 20 | 3.4 | 20 | 20.00 | 94.84 | 58.08 |
| F | 8 | 60.0 | 20 | 3.4 | 5 | 5.00 | 101.78 | 80.07 |
| F | 9 | 60.0 | 20 | 3.4 | 5 | 50.00 | 94.12 | 18.93 |
| F | 10 | 60.0 | 20 | 3.4 | 10 | 5.00 | 96.34 | 583.40 |
| F | 11 | 60.0 | 20 | 3.4 | 10 | 50.00 | 99.13 | 15.04 |


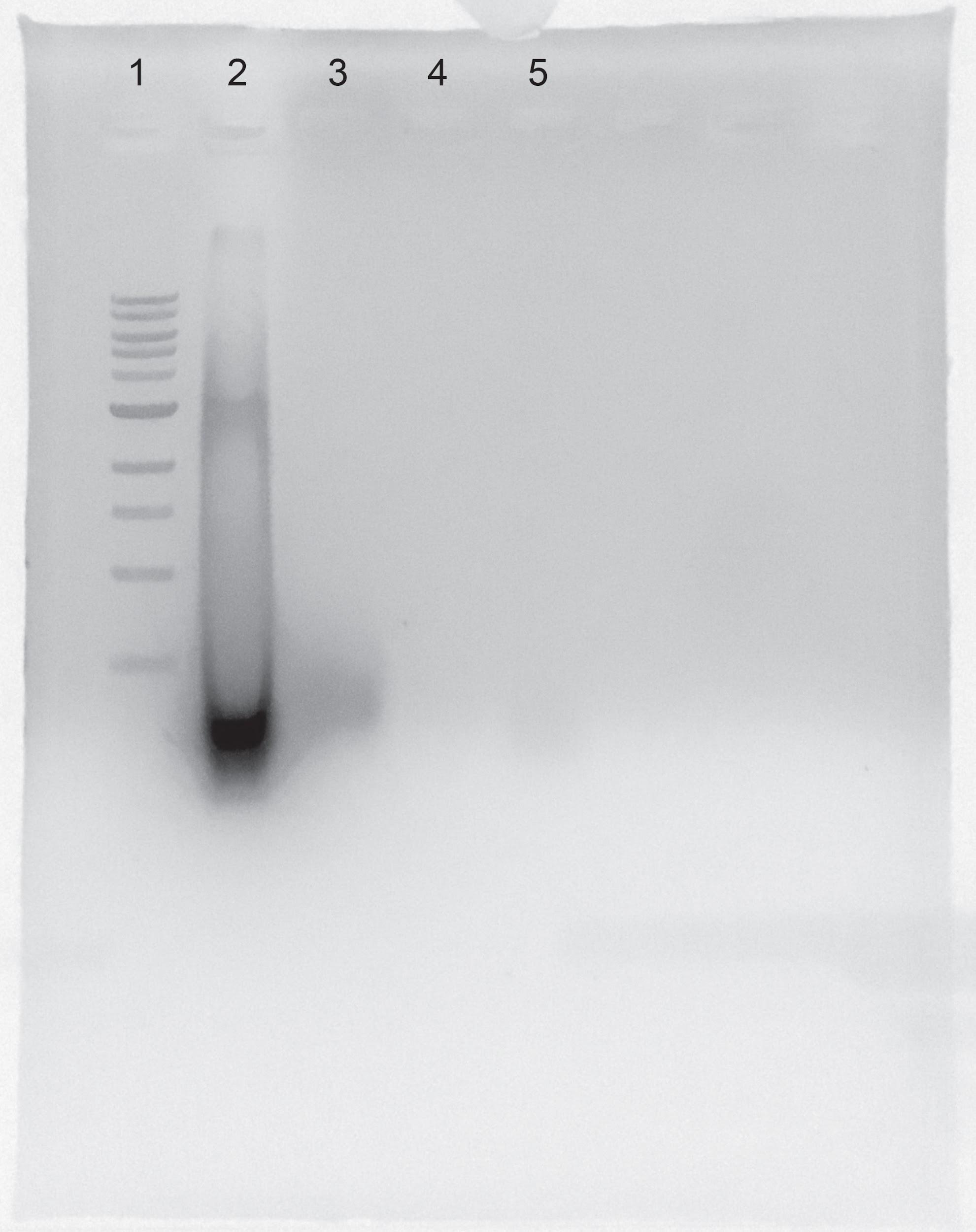


**Supplementary Figure S2: Determination of DNA content in precipitation-purified GRFT samples.** Samples of *E. coli* lysate from before and after precipitation were assayed by 1% agarose gel electrophoresis with Ethidium Bromide staining and imaged under UV illumination. Lane 1: 1 kb DNA Ladder (New England Biolabs, Ipswich, MA), 5 μL. Lane 2: *E. coli* lysate immediately after sonication; approximately 450 μg total protein loaded, including approximately 45 μg GRFT. Lane 3: supernatant following the precipitation step; approximately 20 μg GRFT loaded. Lanes 4 and 5: precipitation supernatant following buffer exchange using an Amicon Ultra 10K filter (Millipore Sigma, Burlington MA; total spin time approximately 1 hr at 14000 RCF; >90% recovery expected for DNA >50 bp per manufacturer information). Buffer-exchanged sample lanes contain approximately 18 μg GRFT. Based on an estimated DNA load of 180 μg in the lysate and a limit of detection for Ethidium Bromide of approximately 5 ng DNA, we estimate a GRFT-DNA separation factor >14400 for the precipitation step.

**Supplementary Figure S3.** Characterization of 6x-His-GRFT via SPR. As a positive control for the measurement of precipitation-purified GRFT activity by SPR, a similar analysis was conducted using purified 6x-His-tagged GRFT obtained from Barry O’Keefe at the NIH. See Materials and Methods for detailed methods. Data (n = 1) were analyzed
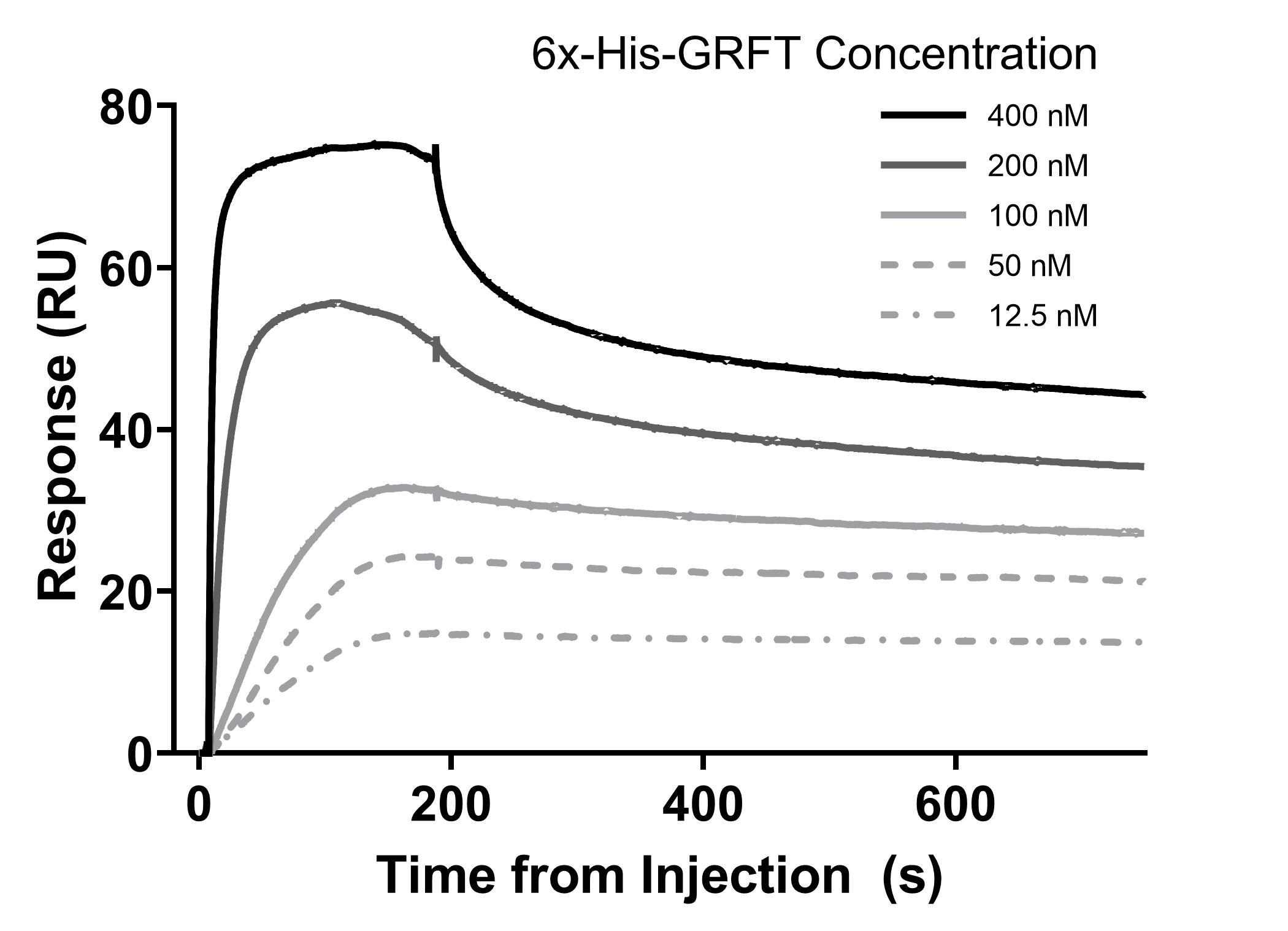
by fitting with a heterogeneous ligand model. Association rate = 1.6 * 10^6^ M^-1^ s^-1^, dissociation rate = 1.5 * 10^-2^  s^-1^, affinity = 9.6 * 10^-9^ M.


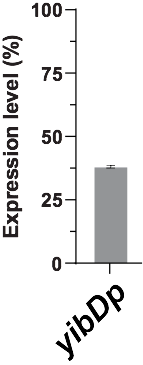


**Supplementary Figure S4.** Expression of Q-GRFT in DLF_0025, in microfermentations in AB media. Gene expression is driven by the low phosphate inducible yibDp promoter.

**References**

1. [Moreb, E. A. *et al.* Robustness testing and scalability of phosphate regulated promoters useful for two-stage autoinduction in E. coli. doi:](http://paperpile.com/b/hGrzFS/zZHqf)[10.1101/2020.01.26.920280](http://dx.doi.org/10.1101/2020.01.26.920280)[.](http://paperpile.com/b/hGrzFS/zZHqf)

2. [Li, S., Ye, Z., Lebeau, J., Moreb, E. A. & Lynch, M. D. Dynamic control over feedback regulation improves stationary phase fluxes in engineered E. coli. *bioRxiv* 2020.07.26.219949 (2020) doi:](http://paperpile.com/b/x0mOul/sJ0L)[10.1101/2020.07.26.219949](http://dx.doi.org/10.1101/2020.07.26.219949)[.](http://paperpile.com/b/x0mOul/sJ0L)

3. [Alam, A. *et al.* Technoeconomic Modeling of Plant-Based Griffithsin Manufacturing. *Front Bioeng Biotechnol* **6**, 102 (2018).](http://paperpile.com/b/hGrzFS/wbp9)

4. [Farid, S. S. PROCESS ECONOMIC DRIVERS IN INDUSTRIAL MONOCLONAL ANTIBODY MANUFACTURE. in *Process Scale Purification of Antibodies* (ed. Gottschalk, U.) vol. 23 445–466 (John Wiley & Sons, Inc., 2017).](http://paperpile.com/b/hGrzFS/Jef5)

5. [Qi, W., Sathre, R., Morrow, W. R., III & Shehabi, A. *Unit price scaling trends for chemical products*.](http://paperpile.com/b/hGrzFS/909J) <https://www.osti.gov/servlets/purl/1236367> [(2015).](http://paperpile.com/b/hGrzFS/909J)

6. [NATIONAL AGRICULTURAL STATISTICS SERVICE. *AGRICULTURAL STATISTICS 2019*.](http://paperpile.com/b/hGrzFS/qifj) <https://www.nass.usda.gov/Publications/Ag_Statistics/2019/2019_complete_publication.pdf> [(2019).](http://paperpile.com/b/hGrzFS/qifj)

7. [Neidhardt, F. C. & Curtiss, R. *Escherichia coli and Salmonella : cellular and molecular biology*. (ASM Press, 1996).](http://paperpile.com/b/hGrzFS/D1iP)

8. [Wang, X., Hunter, A. K. & Mozier, N. M. Host cell proteins in biologics development: Identification, quantitation and risk assessment. *Biotechnol. Bioeng.* **103**, 446–458 (2009).](http://paperpile.com/b/hGrzFS/o3oV)

9. [World Health Organization. *Requirements for the use of animal cells as in vitro substrates for the production of biologicals (requirements for biological substances no. 50). In: WHO Expert Committee on Biological Standardization. Forty-seventh report*. (1998).](http://paperpile.com/b/hGrzFS/jVdS)

10. [He, X. & Snyder, M. *A Purification Strategy for Clinical-Grade Monoclonal Antibody Using Hydrophobic Cation Exchange Chromatography*.](http://paperpile.com/b/hGrzFS/VeNs) <http://www.bio-rad.com/webroot/web/pdf/ps/literature/Bulletin_6241A.pdf>[.](http://paperpile.com/b/hGrzFS/VeNs)

11. [Bonnerjea, J., Oh, S., Hoare, M. & Dunnill, P. Protein Purification: The Right Step at the Right Time. *Biotechnology* **4**, 954–958 (1986).](http://paperpile.com/b/hGrzFS/sDG2Q)

12. [Chen, R. H. *et al.* Factors affecting endotoxin removal from recombinant therapeutic proteins by anion exchange chromatography. *Protein Expr. Purif.* **64**, 76–81 (2009).](http://paperpile.com/b/hGrzFS/JI7VS)

13. [Haber, C., Skupsky, J., Lee, A. & Lander, R. Membrane chromatography of DNA: conformation-induced capacity and selectivity. *Biotechnol. Bioeng.* **88**, 26–34 (2004).](http://paperpile.com/b/hGrzFS/zcrNo)

14. [Steckel, H., Eskandar, F. & Witthohn, K. Effect of excipients on the stability and aerosol performance of nebulized aviscumine. *J. Aerosol Med.* **16**, 417–432 (2003).](http://paperpile.com/b/hGrzFS/cECm)

15. [Albasarah, Y. Y., Somavarapu, S. & Taylor, K. M. G. Stabilizing protein formulations during air-jet nebulization. *Int. J. Pharm.* **402**, 140–145 (2010).](http://paperpile.com/b/hGrzFS/wmEA)
